# Similarity in Shape Dictates Signature Intrinsic Dynamics Despite No Functional Conservation in TIM Barrel Enzymes

**DOI:** 10.1371/journal.pcbi.1004834

**Published:** 2016-03-25

**Authors:** Sandhya P. Tiwari, Nathalie Reuter

**Affiliations:** 1 Department of Molecular Biology, University of Bergen, Pb. 7803, Bergen, Norway; 2 Computational Biology Unit, Department of Informatics, University of Bergen, Pb. 7803, Bergen, Norway; Iowa State University, UNITED STATES

## Abstract

The conservation of the intrinsic dynamics of proteins emerges as we attempt to understand the relationship between sequence, structure and functional conservation. We characterise the conservation of such dynamics in a case where the structure is conserved but function differs greatly. The triosephosphate isomerase barrel fold (TBF), renowned for its 8 β-strand-α-helix repeats that close to form a barrel, is one of the most diverse and abundant folds found in known protein structures. Proteins with this fold have diverse enzymatic functions spanning five of six Enzyme Commission classes, and we have picked five different superfamily candidates for our analysis using elastic network models. We find that the overall shape is a large determinant in the similarity of the intrinsic dynamics, regardless of function. In particular, the β-barrel core is highly rigid, while the α-helices that flank the β-strands have greater relative mobility, allowing for the many possibilities for placement of catalytic residues. We find that these elements correlate with each other via the loops that link them, as opposed to being directly correlated. We are also able to analyse the types of motions encoded by the normal mode vectors of the α-helices. We suggest that the global conservation of the intrinsic dynamics in the TBF contributes greatly to its success as an enzymatic scaffold both through evolution and enzyme design.

## Author Summary

Proteins are dynamic entities, and their flexibility is intimately linked with function. Some suggest that function drives the conservation of flexibility in proteins, while it has also been observed that proteins with similar structures also exhibit similar flexibility. To investigate the role of shape vs. function in the conservation of protein flexibility, we studied proteins with the TIM Barrel Fold, a common enzyme scaffold, from five functionally distinct protein families, computationally. The results from our comparative analysis approach show that the similarity in structural fold dictates overall similarity in intrinsic flexibility between the proteins. In particular, the dynamic characteristics TIM Barrel Fold allow it to accommodate a variety of differences in enzymatic active site positioning without disrupting its overall flexibility. This study has an implication in the way we understand the evolvability of protein sequence and structure in nature. In addition, the signature patterns of flexibility we describe should be considered when designing novel enzymes with the same fold.

## Introduction

Understanding a proteins’ inherent flexibility, or intrinsic dynamics, is fundamental to understanding the mechanism with which they are able to perform their function. Yet we know little about the conservation of dynamic properties in a structural fold, whether the similarity is due to or regardless of evolutionary conservation. In many cases, protein families can be distinguished by their similarity in dynamics [1, 2], however there is also growing evidence that this may be influenced by the level of similarity in their overall structural topology, which can be robust to mutations [3, 4]. General properties have been ascribed to elements of protein structures, such as the correlated motions of β-sheets [5], while a clear similarity between the flexibilities of non-homologous enzymes catalysing the same reaction has also been demonstrated [6]. The role of dynamics is not limited to the most flexible regions of the protein; the most rigid regions of proteins have been suggested to act as energy sinks as part of their functional role [7]. Studies by Micheletti and colleagues have also suggested that dynamics is conserved for function, regardless of the structural conservation [8, 9].

Enzymes that possess the TIM Barrel Fold (TBF), named after the enzyme triosephosphate isomerase (TIM), provide a good case for exploring the intrinsic dynamics of structures that are similar in shape yet completely different in function. In a comprehensive review by Nagano *et al*. in 2002, TBF proteins were described with regards to structural similarity as domains and functional abilities [10]. They found that only four out of the 21 protein families analysed were found to have direct sequence-based evolutionary relationships. Due to its property of being very evolvable [11], TBF is a popular fold in the field of protein design [12–14]. The functions of these proteins span five out of six of the Enzyme Commission classes, another reason it earns the distinction of being a “super-fold”[15].

**Fig 1:**
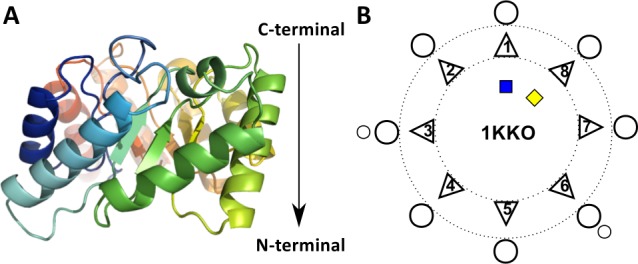
Schematic representation of the TIM Barrel Fold topology. (A) A cartoon representation of a TBF domain structure, from methylaspartate ammonia lyase (PDB 1KKO). The sequence consists of 8 β-strand-α-helix repeats that form a barrel-like structure. This barrel possesses directionality in terms of a C-terminal end, represented at the top of the structure here and the N-terminal end at the bottom, indicated by the arrow. (B) The two-dimensional secondary structure element arrangement as viewed from the top of the C-terminal end of the enzyme. The diversification of the fold occurs with the addition of secondary structure elements, typically at the C-terminal end, indicated here by small circles. The triangles represent the β-strands, the circles represent the α-helices, and the blue and yellow squares are the N- and C-termini respectively. The dotted lines represent the circular spatial arrangement of the TBF, such that the β-strands are able to close and form a parallel β-barrel core, flanked by their α-helices.

The TBF consists of eight β-strands and α-helices that alternate in sequence and fold into a barrel-like shape (Fig 1). The eight β-strands close to form a parallel β-barrel core, while the eight α-helices surround this core, each flanking its corresponding β-strand. The TBF is the only reported case of the parallel β-barrel within its structural topology, as most β-barrels are anti-parallel and possess a variety of shear numbers (a descriptor of the inter-strand twisting) [16–18]. The diversity of the TBF is accommodated by the loops between the eight β-α pairs that are able to accommodate additional secondary structure elements (SSEs) or domains in some proteins [10, 14, 19]. TBF proteins can exist as part of multi-domain enzymes, displaying additional versatility as a scaffold for many reaction types.

Aside from the similarity in shape, enzymes with this fold have other common traits. For instance, most of their active sites occur at the C-terminal end of the barrel and can be placed on different secondary structures and most commonly on the loops between them, providing a greater number of possibilities for their positioning in sequence. For example, triosephosphate isomerase has catalytic residues on β-strand 1 and loops 1, 4 and 6, while chitinase B has them on the β-loop-α unit 4 and loop 6 (S1 Fig, panels 1N55 and 1E15). In general, all of the catalytic sites are found on the C-terminal end of the barrel-like structure, which is referred to as the catalytic end, while the N-terminal end is referred to as the stability end (Fig 1) [19]. The β-barrel core of the TBF proteins is mostly hydrophobic [18], where the amino acids are found to be rather packed, preventing access to solvent. A mathematical study on the structural features of parallel β-barrels by Lasters *et al*. [20] showed that eight β-strands form the optimal configuration for the packing of amino acid side chains inside the β-barrel core. They found that the parallel barrels that make up the protein core appear to tolerate little variability on the right-handedness of its shear and its stability through inter-strand hydrogen-bonds, resulting in the conservation of key geometric parameters such as inter-strand twist and strand number.

Despite all the characterisation of the fold via experiments and structural bioinformatics methods, the understanding of the dynamics has been restricted to the flexibility of the substrate binding site loops of the TIM enzyme, e.g. via solid-state nuclear magnetic resonance [21], molecular dynamics simulations [22] and normal mode analysis (NMA) using elastic network models (ENM) [23]. This fold has also been used as a test case for protein engineering and co-evolution studies [11, 12, 24].

Intrinsic dynamics as described by ENM-based NMA calculations is a quick and reliable measure of protein dynamics and flexibility, especially at the secondary structure and domain levels. ENM-based NMA has been particularly amenable to the comparison of intrinsic dynamics of multiple structures [4, 25]. Despite evidence that there is conservation of dynamics that follows the conservation of sequence and structure, it has been a challenge to separate this from the influence of structural topology independent of evolutionary relationships. Others have also studied families and superfamilies of proteins with low sequence conservation using ENM-based NMA, showing its relevance and usefulness when exploring their similarities [1, 2, 8, 26–34].

Knowing detailed attributes of the TBF only drives our curiosity as to its success as a common structural framework further, regardless of the mode of its structural and sequence conservation. We seek to explore the similarities in intrinsic dynamics using ENM-based NMA between five structures from five different superfamilies with varying levels of structural similarity and evolutionary relationship, as revealed by Nagano *et al*. [10]. Focussing on the role of the secondary structures and their flexibility within the context of the fold, we show that the intrinsic dynamics can be better compared when including homologous proteins from within the five superfamilies. We relate the catalytic and ligand sites of these proteins to their rigidity, as defined by the fluctuation profiles. To characterise the differences in rigidity, we examine the significant correlated movements of the secondary structure elements from five representative TBF structures from each superfamily. Finally, we also characterise the types of displacements of the β-barrel core and the outer α-helical bundle undergo. Upon finding that the α-helices are more mobile, collectively and independently, than the β-barrel core, we characterised the displacements of the individual α-helices as well.

## Results

Our dataset consists of five proteins listed in Table 1 and covering four of the six enzymes classes according to their Enzyme Commission (EC) number. They belong to five different superfamilies according to the classification from Nagano *et al*. [10]: Triose phosphate isomerase (TIM), Aldolase class I (ALD1), Enolase (ENOL), Chitinase (CHTN) subfamily of the Glycosidases (GLYC) and Phosphatidylinositol (PI) phospholipase C (PIPLC). The dataset is further described in the Methods section and in the S1 Table.

**Table 1:** Proteins dataset: List of the five TBF structures selected with their structural classification and functional annotation.

Enzyme	CATH ID	PDB ID	E.C. number
	**Superfamily name**		
Triosephosphate isomerase	3.20.20.70	1N55	5.3.1.1
	Aldolase Class I		
Chitinase B	3.20.20.80	1E15	3.2.1.14
	Glycosidase		
Methylaspartate ammonia lyase	3.20.20.120	1KKO	4.3.1.2
	Enolase		
Transaldolase B	3.20.20.70	3CWN	2.2.1.2
	Aldolase Class I		
Glycerophosphodiester phosphodiesterase	3.20.20.190	3CH0	3.1.4.46
	Phosphatidylinositol phosphodiesterase		

### The influence of multiple structural alignments on comparative measures

To assess the overall similarity in intrinsic dynamics between structures, we perform analysis that is reliant on a multiple structure alignment. The flexibility is compared at the amino acid positions that are comparable within the set of structures i.e. the ones that can be structurally aligned through the whole dataset. The actual calculation of the dynamical similarity score is influenced by the alignment of the structures considered due to the alignment’s role in defining the comparable positions within a set of proteins [25]. When comparing structures that have poor sequence identities ranging from 18% to 45% (S3 Fig, left panel), such as these five TBFs (with PDB ID.s 1N55, 1E15, 1KKO, 3CH0 and 3CWN, Table 1), constructing a reliable multiple structure alignment can be challenging. This challenge is compounded in structures with a TBF due to the symmetry of their fold, and difference in sizes, especially with regards to volume [35].

**Fig 2:**
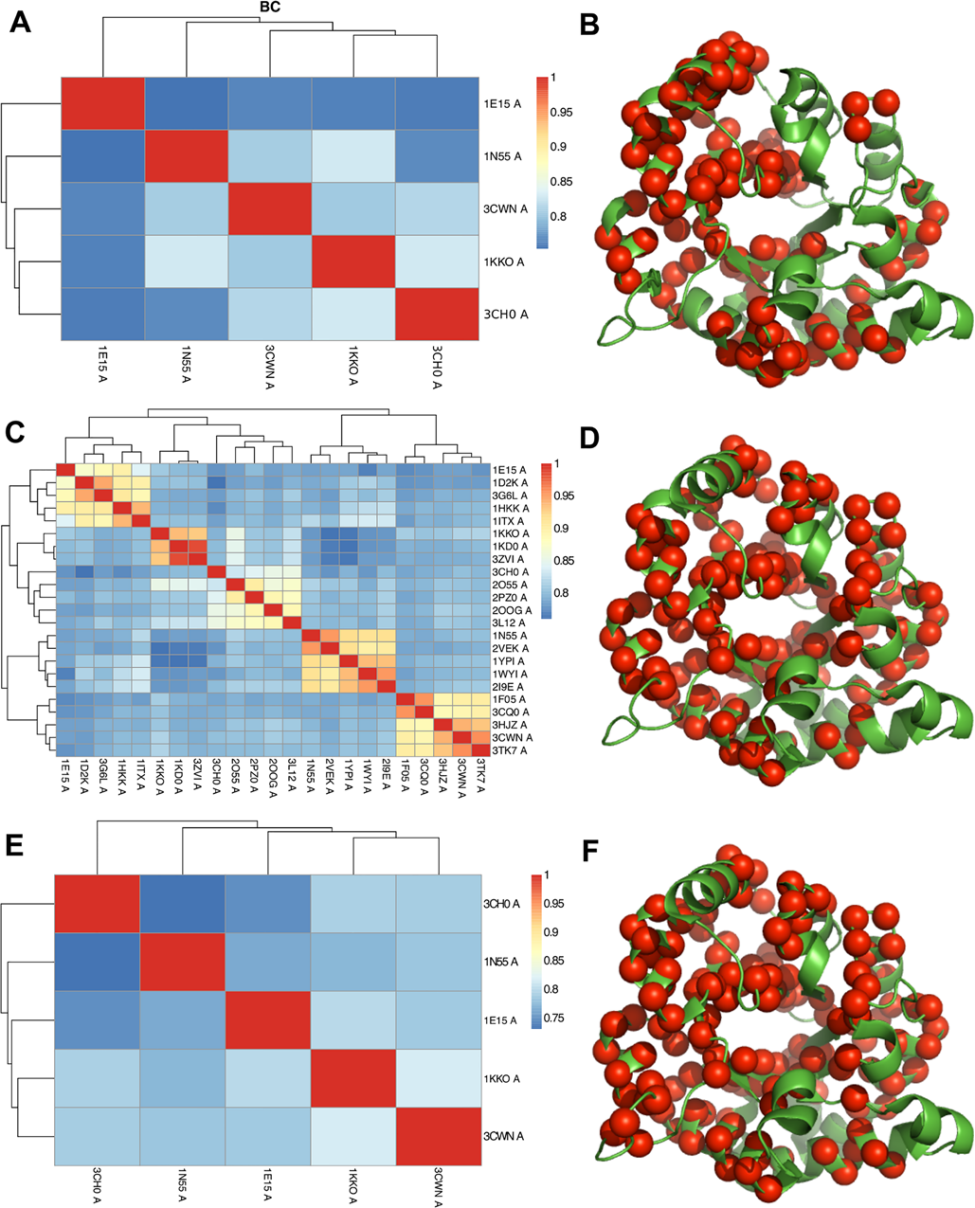
Bhattacharyya coefficient (BC) scores and conserved amino acid positions in the structural alignments. Heatmap representations of the pairwise BC scores for the structural alignments performed on (A) the five representative TIM barrel fold structures, (C) the 23 homologues and (E) the main five structures as extracted from the alignment of 23 homologues. These alignments correspond to (B) 106, (D) 135 and (F) 145 Cα atom positions conserved (red spheres), respectively. Structures are labelled by their PDB identifiers and represented with green cartoons highlighting their secondary structure elements. The colour scale on the BC maps goes from blue to yellow to red, for low (~0.75) to high (1.0) BC scores. The dendrogram reflects the hierarchical clustering based on the BC scores.

Here, we used MUSTANG[36] to obtain multiple structural alignments of the proteins in the dataset and we use a global similarity measure, the Bhattacharyya coefficient (BC) score (See Methods section), not just as a measure of similarity between the intrinsic dynamics of the structures but also as a means to validate the quality of each alignment. We recently demonstrated that this strategy was reliable for a dataset of 53 structures with the TIM-barrel fold [25]. When aligning the five main structures, we find that the first α-helix of 1N55 shifts by one α-helical unit to correspond to the second α-helical units of the other four structures, causing a mismatch. The mismatch results in the loss of conserved points between the first and eighth strands of the structural alignment, which are not considered in the calculation of the BC score (S2 Fig). The BC analysis between the five structures has scores that range from 0.75 to 0.83, with the least similar pair of structures being 1N55 and 1E15 (0.75) and the most similar pairs being 1KKO & 1N55 and, 1KKO & 3CH0 (0.83 for both pairs) (Fig 2A). In this alignment, 106 residues are conserved in the alignment (Fig 2A).

Incorporating evolutionary information by using multiple sequences is a usual practice that adds robustness to sequence alignments. Following this principle, we performed a multiple structural alignment with 23 structures (Fig 2 and S2 Table), consisting of five homologues per superfamily, except for 1KKO where only three were found based on our criteria (cf. Methods, S3 Fig—right panel). In this alignment, 135 evenly distributed positions are conserved (Fig 2D). The clustering of the BC scores showed that the structures group according to their superfamilies (Fig 2C). Moreover, we see that the two former separate superfamilies belonging to the structures 1N55 and 3CWN have clusters that are next to each other. 1N55 and 3CWN currently belong to the same Aldolase Class I in CATH (cf. Methods). The scores between the five main structures range from 0.78 to 0.82, whereas the scores within the homologous sets tend to be between 0.84 to 0.95 for 1E15, 0.90 to 0.95 for 1N55, 0.83 to 0.90 for 3CH0 and 0.89 to 0.96 for 3CWN. The scores show a separation between the structures within and between the superfamilies and correspond to the separation seen in Fuglebakk *et al*. [37]. The clustering of the 23 structures according to their BC scores reproduces the classification by Nagano *et al*. [10].

Next, we extracted the alignment of the five main structures from the alignment of the 23 structures, which naturally increased the number of conserved points by ten. Thus, 145 residue positions were considered in the BC score calculation here (Fig 2F). Despite using the exact same structural alignment as the previous one, a mere increase in ten conserved positions in the alignment reorders them in the clustering (Fig 2E). This results in an incorrect grouping of the two enzymes from the Aldolase Class I superfamily (1N55 and 3CWN).

Thus, we find that the structural alignment and BC calculations with 23 structures are more reliable than the ones obtained from just the five main structures. Incidentally, even if we artificially increase the number of corresponding points on the structures, we are unable to obtain the correct ordering of the structures. This could be due to the loss of evolutionary information when we allow parts of the structures that do not naturally align to be considered in the comparison.

### The inner β-barrel of the fold forms a rigid core independent of their flanking α-helices

**Fig 3:**
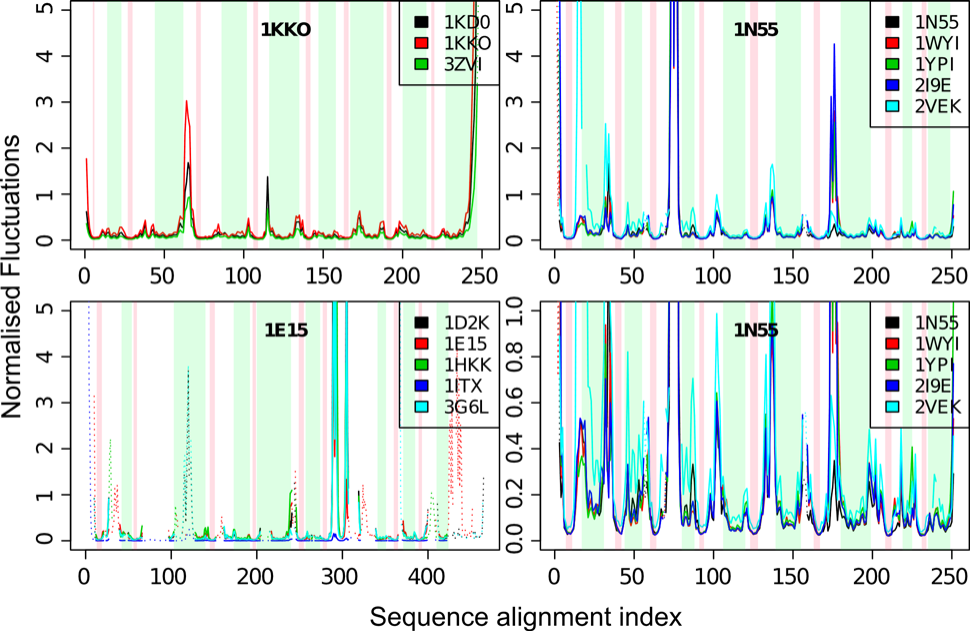
Normalised fluctuations of three out of the five TIM superfamilies, 1KKO, 1N55 and 1E15, and their orthologues. Green bars delimitate α-helical regions, while red bars correspond to the β-stranded regions. The fourth panel (bottom right) is a zoomed in profile of 1N55.

The normalised fluctuations show the magnitude of mobility of each Cα atom, and are often referred to as *theoretical B-factors* of the structures. When examining the normalised fluctuations profiles for the five representative proteins (Fig 3 and S4 Fig), we find that the β-strands and the α-helices of the TBF are immobile compared to the regions in-between them. Between the β-strands and the α-helices of the TBF, we find that the β-strands fluctuate less, whereas the α-helices show greater tendency for fluctuation. Yet, as we expected, both of these secondary structure elements fluctuate much less than the loop regions between them. The only exception is the second helix in 1E15, where two of the homologues (1D2K, 3G6L) possess large fluctuations within the secondary structure region. Upon closer scrutiny, we find that the α-helices in these two homologues are shorter, and that segments of the secondary structure with large fluctuations are disordered in those structures while they are α-helical in others.

**Fig 4:**
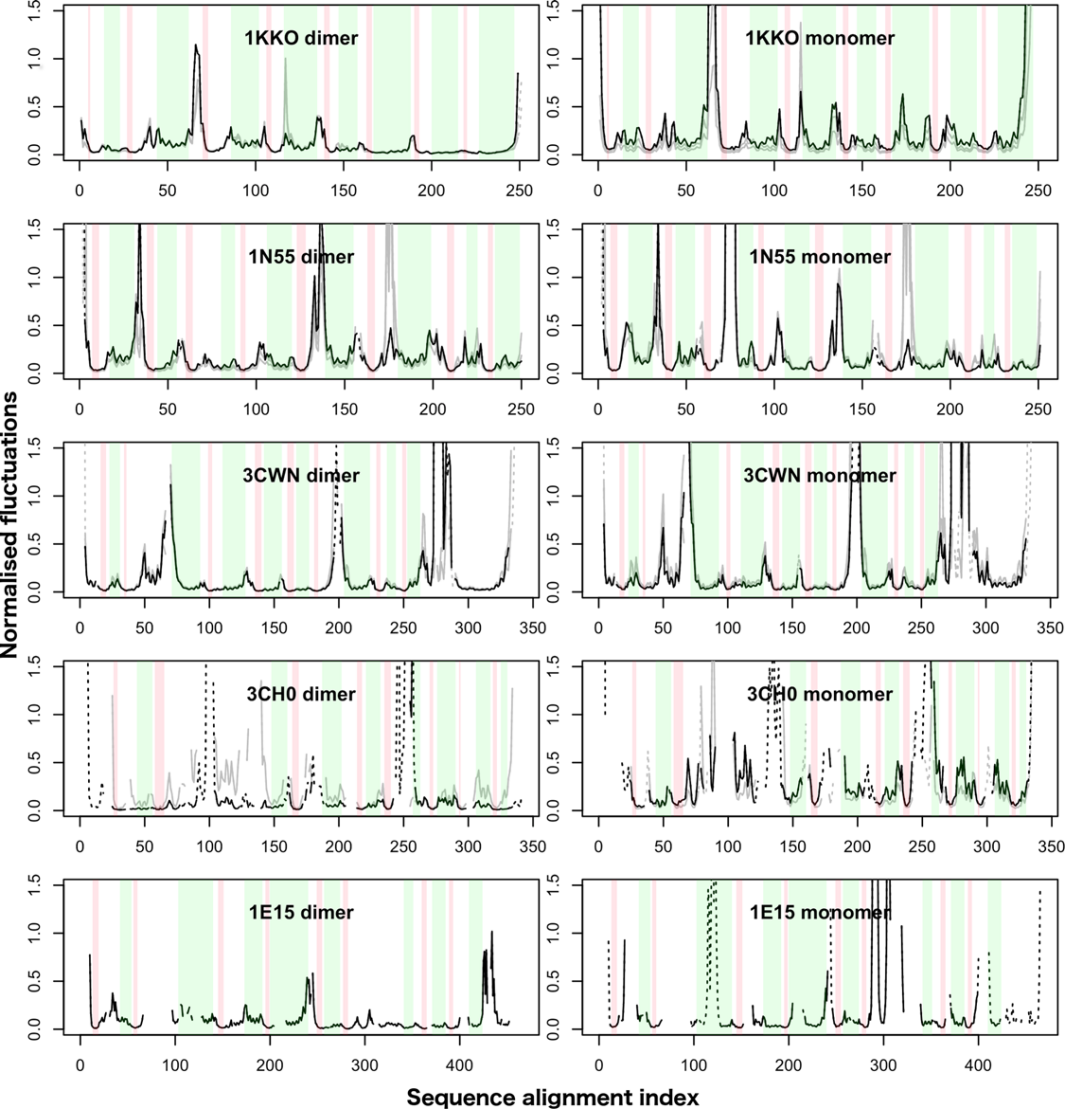
Normalised fluctuation profiles of dimer and monomer forms of the five main enzymes (black) and their orthologues (grey). In both panels, only the profiles of the TBF from chain A are displayed, following the same residue index as the monomer. Green bars indicate α-helices, while the red bars indicate β-strands. Only some of the enzymes in the dataset form dimers, and the orthologues included in the normalised fluctuations profiles of the dimer forms are shown for structures listed in S3 Table.

We compared the normalised fluctuations profiles of the TBF domains in Fig 3 to the profiles calculated from ENMs with the accompanying subunits or domains according to their biological assemblies (Fig 4). We observe that the β-strands remain the most rigid parts of the TBFs, while the α-helices are slightly more flexible. The main difference between the profiles lies in the loop regions and we find that oligomerisation and the presence of other domains mainly act to rigidify loop regions, or modulate the rigidity of these loops, without changing the rigidity profiles of the secondary structures significantly.

**Fig 5:**
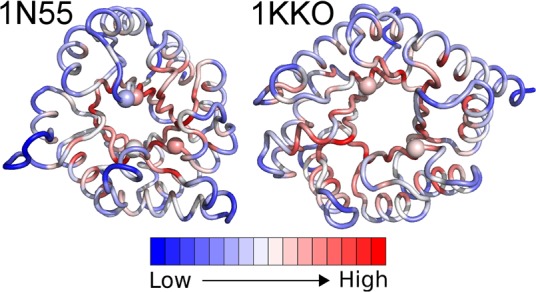
Normalised deformation energies (calculated over all non-trivial normal modes) for 1N55 and 1KKO. Scale ranges from low (blue) to intermediate (white) to high (red) normalised values of the deformation energies. The spheres represent the positions of the catalytic residues.

The trend of rigidity is further illustrated by the normalised deformation energies, as displayed on the structures themselves in Fig 5 and S5 Fig. The normalised deformation energies calculated here inform about the local flexibility of each Cα atom relative to its neighbouring atoms [38]. We find that the β-barrel core has high deformation energies, while the loop regions and surface residues have lower energies. These differences indicate that the β-barrel core acts as a rigid anchor of the structure, in contrast to the slightly more mobile α-helices. This also corresponds to the fluctuation analysis (Fig 3 and S4 Fig), which shows that the generally hydrophobic cores of these structures are robust to fluctuations.

**Fig 6:**
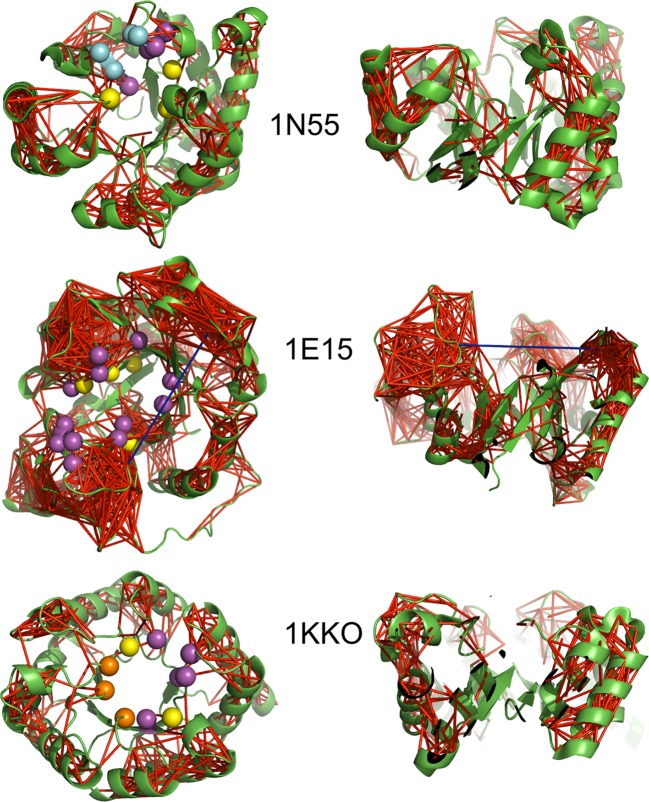
Distribution of distant significant correlations in 1N55, 1E15 and 1KKO. Top views (left) from the perspective of the C-terminal end and side views (right) with the N-terminal end at the bottom and the C-terminal end at the top. Structures are displayed with the cartoon representation in green. Sticks are drawn between each pair of residue positions that are at least 8 Å apart and have significant correlations (cf. Methods). The sticks in red indicate positive correlations above the score threshold at the 95^th^ percentile rank of the absolute values of the correlations and in blue indicate negative values of correlations below the negative of the score threshold. The yellow spheres represent the positions of the catalytic amino acids while the purple, cyan and orange spheres represent substrate, phosphate and metal-ion binding residues, respectively.

The correlations between pairs of residues in the protein inform us about the coupling of the motions across regions, SSEs or domains of proteins. To capture significant correlations between distant amino acids, we considered pairwise correlation scores above the 95^th^ percentile rank, for Cα atoms that are at least 8 Å apart (cf. Methods, as implemented in [39]). We found that the bulk of these strong correlations lie within the loop regions, connecting to and spanning the helices (Fig 6). When examining 1KKO, the structure with the least number of accessories on the periphery of the TIM barrel, we see that these strong and distant correlations tend to be uniformly distributed around the barrel, connecting to the strands via the loops above and below them. The density of these correlations at the loop regions also changes with the presence of accessory secondary structure elements.

The same analysis was performed on the biological assemblies to explore the impact of the oligomerisation on the distribution of the significant, distant correlations. We found that most of the correlations lie in the loop regions and within the α-helices, while the β-strands are correlated to the α-helices via the loop regions (e.g. 1N55, S9 Fig). This pattern is highly similar to the one we observe in the monomeric forms in Fig 6. Moreover, there is a densification of significant distant correlations in regions that are away from the oligomeric interface, reflecting the rigidification of the loops upon oligomerisation.

In three structures, 1E15 (Fig 6), 3CWN and 3CH0 (S6 Fig), regions with accessory elements also possess strong, distant anti-correlations with other such highly correlated regions, none of which involve the secondary structures of the main fold. Moreover, the β-strands and their flanking α-helices are not correlated together directly, but associate with each other via the loops connecting them. This creates a “gap” in the network of correlations between the β-barrel core and outer flanking helical bundle (Fig 6 and S6 Fig). The exception is in the case of 3CH0, where the β-strand that carries the catalytic residue is well correlated to the connecting α-helix.

### The catalytic sites are located in rigid regions of the fold

**Fig 7:**
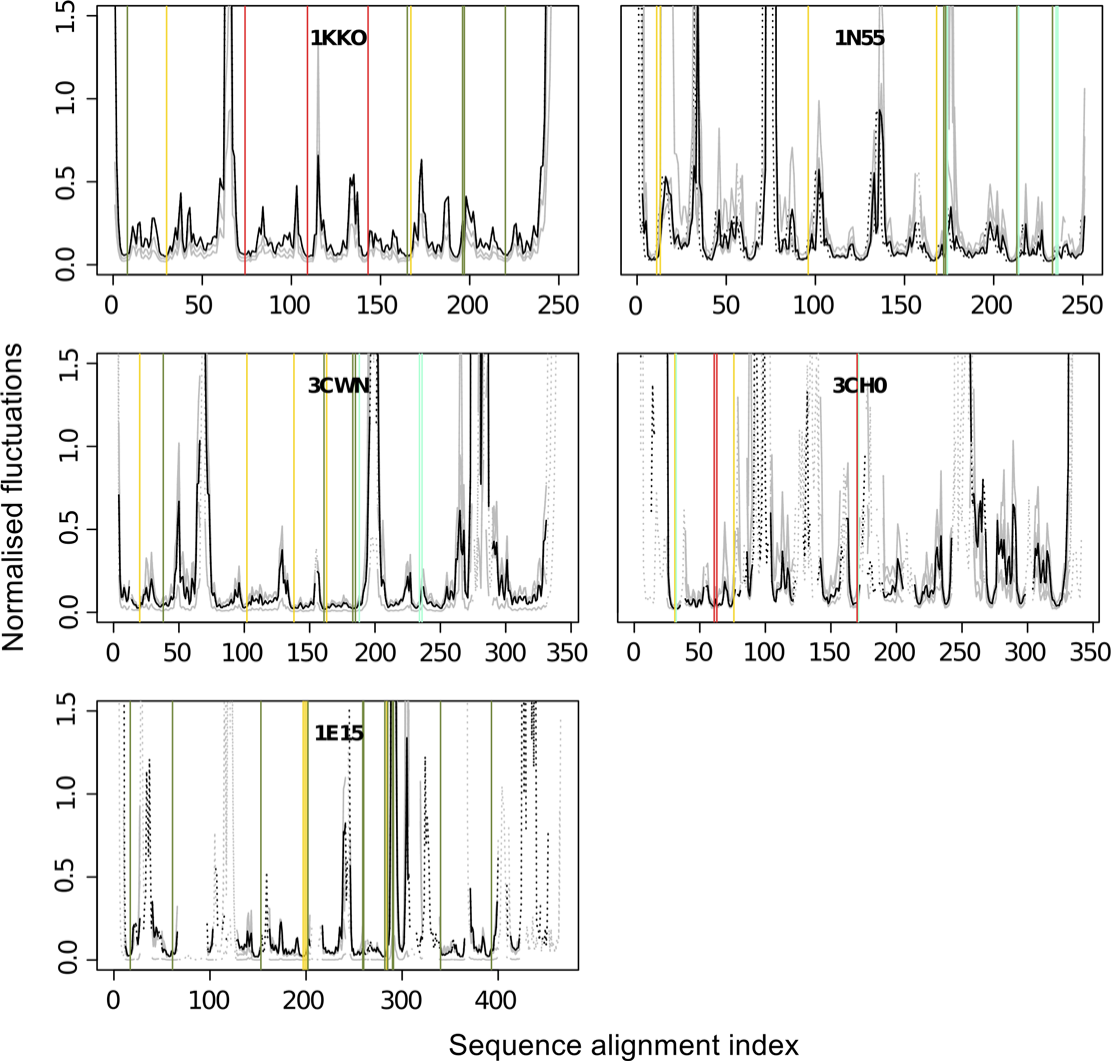
Normalised fluctuations with annotated functional sites. The catalytic residue sites annotated as yellow solid lines, with the substrate binding sites as green, phosphate-binding sites as cyan and the metal ion binding sites as red. The black solid lines represent the main TBF protein for that superfamily, while the grey lines are the homologues. The dotted lines represent the gaps in the alignment.

Like the secondary structure elements, the catalytic residues also correspond to more rigid parts of the structure, as shown in Fig 7. Most substrate-binding, phosphate-binding and metal ion-binding sites also follow this trend, with some exceptions such as the substrate-binding residues W220 and E221 in 1E15 (index 290 and 291 in Fig 7) and T360 and C361 in 1KKO (index 196 and 197 in Fig 7) and the phosphate-binding residue G173 in 1N55 (index 174 in Fig 7). It should be noted that even the exceptions do not lie in the regions with the highest peaks. Despite the extremely low flexibility, the catalytic residues lie in the interface between the least and the most deformable subdomains of the structure (Fig 5). The observation in Fig 5 can be explained by the need for these residues to be in close proximity to the more deformable substrate-binding sites of the enzymes.

As the catalytic residues are located at the C-terminal end of the β-barrel, we isolated the distant, significant correlations shown in Fig 6 that involve the β-strands of the five main TBF enzymes. In doing so, we observed that there were not many of such correlations between the β-strands and within the β-barrel core in comparison with the rest of the structures. Strong correlations in the β-strands are mainly in close range and typically do not span beyond their neighbouring strand. Thus, they do not appear in the distant correlation analysis but only if we decrease the distance threshold to 4 Å that corresponds to the approximate distance between the Cα atoms of two adjacent β-strands (S8 Fig).

**Fig 8:**
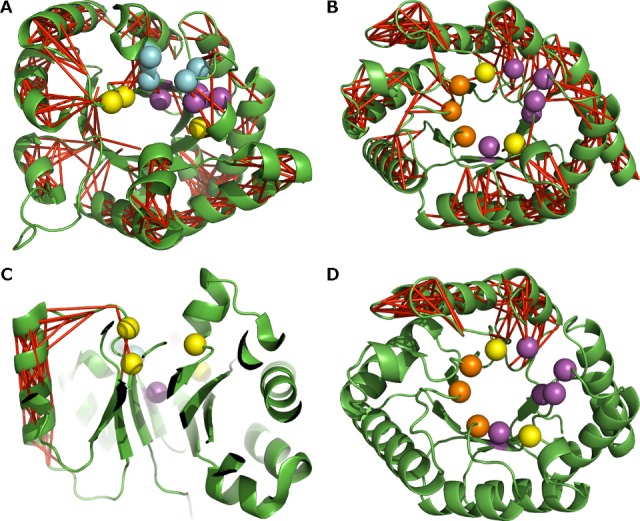
Significant, distant correlations that involve β-strands as observed in 1N55 and 1KKO. In 1N55 (A) and 1KKO (B), the red sticks illustrate the correlations with scores within the 95^th^ percentile and between Cα atoms at least 8 Å apart. Examples of a single continuous network of red sticks as shown in 1N55 (C) and 1KKO (D), illustrate the significant correlations implicated on the first β-strand extending to the α-helix via the loop. The yellow spheres show the active site residue positions. The Cα atoms of catalytic residues are represented as yellow spheres, the substrate binding by purple spheres, the phosphate binding by cyan spheres, and the metal ion binding by orange spheres.

The significant, distant correlations connect to the larger hubs that run along the flanking α-helices via the loops. As a result, we find that the catalytic residues are situated close to these hubs of distantly correlated residues between the strands and following helices, as illustrated in Fig 8A with 1N55. This is generally the case with all the structures to varying degrees. This is seen in a more extended manner in 1KKO (Fig 8B), where we can see that the significant, distant correlations connect the two loops above the C-terminal ends of the β-strands via the secondary structure elements. In the examples of the five main TBFs we analysed, we observe the connection between significant, distant correlations and residues implicated with substrate-, phosphate- and metal-ion binding is less clear than with the catalytic residues (Fig **8**C and **8**D).

### Displacements of the outer α-helical bundles are more favourable than those of the β-barrels

**Fig 9:**
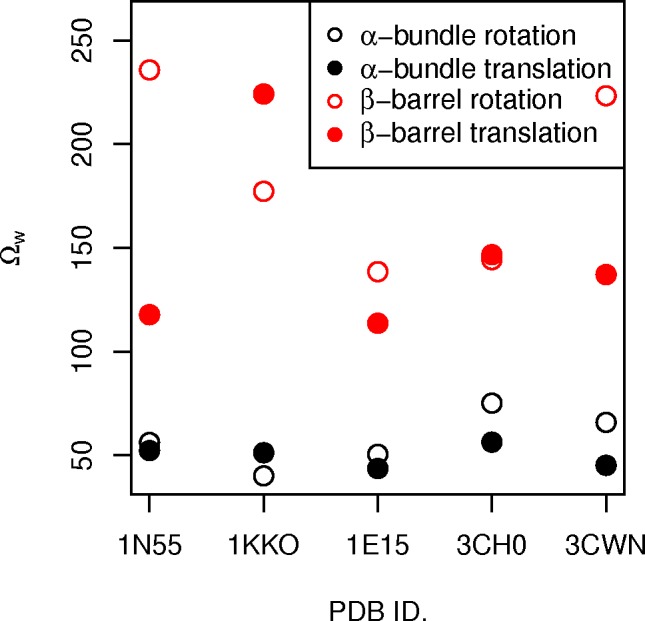
Overlap scores, Ω_*w*,_ of the α-helical helical bundle and β-barrel core normalised ideal displacements, rotation (red) and translation (black), by structure. There is a clear separation in the mobility of the α-helical bundle and the β-barrel core.

A useful way to generalise the contributions of low energy normal modes to relevant domain motions is by finding the overlap between the normal modes and predefined idealised displacement vectors [40]. To determine the propensity for collective displacements of the α-helical bundle and the β-barrel core, we calculated the overlap between the ENM modes and a predefined set of normalised idealised displacements (cf. Methods). The idealised displacement vectors describe translation along and rotation around the axis of inertia of the α-helical bundle or the β-barrel. The overlap is expressed as a score, Ω_*w*,_ which is the sum of the overlap over all the non-trivial modes, weighted by their eigenvalues. This means that Ω_*w*_ values that are low have a greater contribution from lower energy modes (thus more favourable), while Ω_*w*_ values that are high have greater contribution from higher energy modes. As a result, we find that the β-barrels are significantly less mobile than the outer helical bundles, as shown by their much higher Ω_*w*_ scores, confirming the analysis of the normalised fluctuations (Fig 9). Both the rotation and translation displacements of the outer α-helical bundle separate well from the displacements of the β-barrel core for all five structures (Fig 9). Moreover, the translation of the α-helical bundle is more favourable than rotation in four out of the five structures, with varying levels of differences from the rotation displacements.

### Mobile helices are prone to vertical and horizontal displacements regardless of functional amino acid positions

**Fig 10:**
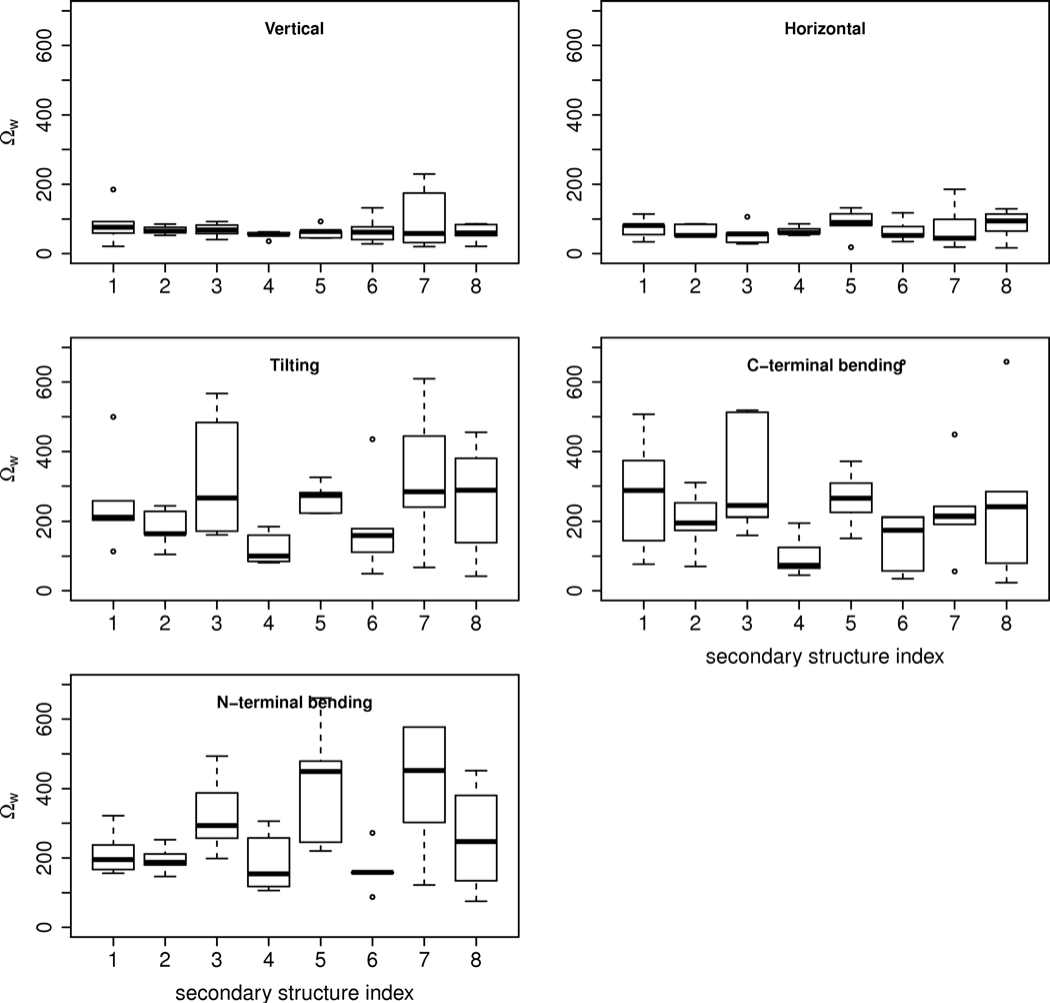
Boxplot of the Ω_*w*_ scores between the normal modes of 1N55, 1KKO, 1E15, 3CWN and 3CH0 and the five normalised ideal vector displacement types defined for individual α-helices. The displacements are shown on Fig 11B. The x-axis refers to the α-helices of the TBF in order of sequence, while the y-axis refers to the Ω_*w*_ scores. The plots are restricted to the range of values from 0 to 700, and exclude an outlier from the N-terminal bending type displacement close to 1000. These scores reflect the overall combination of the overlap with the normal modes weighted by their eigenvalues that reflects their energetic contribution. A low score reflects a preference for the ideal displacement type and vice versa.

We attempted to characterise the types of displacements preferred by the α-helices (Fig 10). We performed overlaps with several normalised ideal vector displacements: vertical, horizontal, tilting, N- and C-terminal bending, and quantified them using the overlap score, Ω_*w*_ (cf. Methods section).

We find that the helices of the five main TBF structures are more prone to vertical and horizontal displacements than tilting and bending (Fig 10). In the vertical and horizontal displacements, we find that helix 7 possesses the greatest range of scores. Of the tilting and bending displacements, the N-terminal bending is least favoured. There also seems to be a preference for the bending of the C-terminal ends of the α-helices, as compared to the tilting and N-terminal bending displacements. As the tilting displacement is a combination of the bending displacement in opposite directions, it is possible that this displacement is less favourable due to the relative immobility of the N-terminal end. Comparing the mobility of the individual SSEs in each structure, we observed that there were no trends that could be related to functional sites (i.e. catalytic residues or ligand binding sites), or the presence of accessory structures. The mobility of the 7^th^ strand of 1E15 can be treated as an artefact from the removal of the domain at the connecting loop. 1KKO is observed to have the most mobile α-helices of all the structures, while in 3CWN the first four α-helices display greater mobility than the last four in sequence when we consider all the displacement types together.

## Discussion

We compared the flexibility of five enzymes with the TIM barrel fold representing four distinct classes in the CATH database. We used an elastic network model for each enzyme to produce normal modes. We then used the Bhattacharyya coefficient (BC) score as a comparative measure for the normal modes and showed that it is able to recapitulate the structural/functional classification of 23 proteins with TBF, including the five chosen representatives. While some elements of the flexibility are specific to each of the five superfamilies, the range of the BC scores shows that the 23 proteins share major flexibility patterns that we discuss below.

### The quality of structure alignment greatly impacts the comparison of overall flexibility

In this study, we have shown that the alignment of the structures improved greatly from the introduction of homologues from each of the five superfamilies. In fact, when performing their study, Nagano *et al*. [10] found that many structures served as “stepping-stone” sequences, which bridged the gaps between distantly homologous sequences. This strategy also had a dramatic impact on the BC scores, which showed a clear separation between the superfamilies, including the clustering of the formerly separated TIM and ALD1 families. As a result, the BC clustering achieved a similar result to Nagano and colleagues [10], possibly with greater ease than their structure and sequence-alignment intensive protocol. The removal of these homologues while preserving the same alignment showed the problem of over-fitting, as we lost the meaningful clustering of the BC scores when ten additional amino acid positions were considered between the five structures. In addition to quantifying the similarity between the structures’ intrinsic dynamics, the BC scores incidentally served as a check for alignment quality, revealing their relevance as a scoring function in multiple structural or structure-based sequence alignments.

Large multiple structural alignments like the ones we have used are not common, beyond examples that demonstrate the efficacy of an algorithm. Efforts made to include dynamical information in alignments are currently only available for pairwise alignments [29]. We emphasise that the comparative analysis of intrinsic dynamics is extremely sensitive to alignments and should be carefully considered when designing comparative flexibility studies and interpreting the results.

### The β-barrel core of the TBF is extremely rigid

The parallel β-barrel core of the TBF is shown to be extremely rigid, relative to the rest of the structure. The flanking α-helices are mobile, but only just, in contrast to the loop regions. These are demonstrated in this study by the combination of the normalised fluctuations profiles (Figs 3 and 4), the deformation energies (Fig 5), and the pairwise correlations (Figs 6 and 7 and 8). The rigidity of the β-barrel is not surprising, considering that the strands are stabilised by an extensive network of hydrogen bonds between them. The influences of the strand-strand interactions are recapitulated by the network of short-range (over 4Å) correlations that originate from the β-barrel core, as shown in S4 Fig. This is unlike anti-parallel β-barrels, which despite being rigid cylinder-like structures, undergo motions such as breathing (that involve the change in cylinder volume), bending and twisting to be able to perform their function [41].

**Fig 11:**
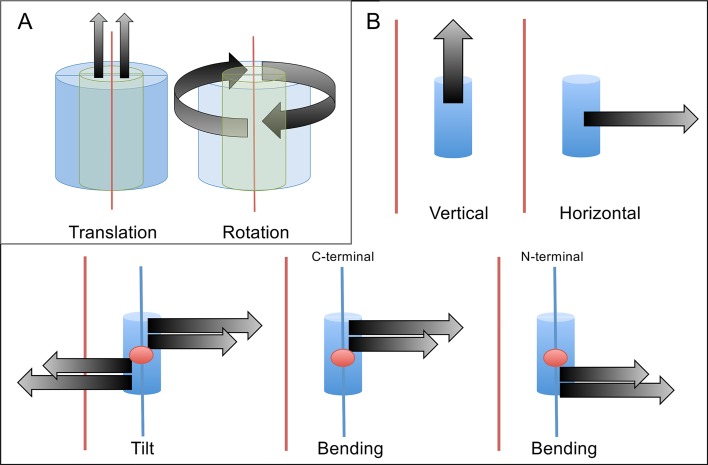
Scheme of the defined normalised ideal vector displacements. (A) Scheme of the translation and rotation of the β-barrel core (green), or the α-helical bundle (blue); the rotation (right) or the translation along the principal axis of inertia of the β-barrel/α-helical bundle (red line, left). The top of the cylinder is analogous to the C-terminal end of the TBF while the bottom of the cylinder is analogous to the N-terminal end. (B) Scheme of vertical, horizontal, tilt and bend (the C- and N-terminal ends) idealised vector displacements of individual α helices (blue cylinder). The vectors are represented as thick black arrows and are defined with respect to the principal axes of inertia for the barrel (red) and of the relevant helix (blue line), as well as to the centre of mass of the helices (red dots).

In contrast, the α-helices display collective movements that are absent in the core of the TBF, as demonstrated by their relative immobility as a collective unit (Fig 9). Our results show that the α-helical bundles prefer to rotate and translate vertically, compared to the β-barrel core. Moreover, the individual α-helices are more likely to translate vertically along their own principal axis of inertia and horizontally away from the centre of mass of the structure by a large extent over tilting and bending (Figs 10 and 11). We also saw that the C-terminal ends of the α-helices seem to displace more than the N-terminal ends. This is consistent with the idea that the generally shorter loops of the N-terminal end serve to provide stability (as seen in the TIM enzyme) and possibly influence mobility of the α-helix [14]. The lack of trend between the mobility of individual helices and the position of the catalytic residues suggests that the flexibility of the helices is dependent on structure. We further suggest that the mobility could have a different role in the functionality of the enzyme; the α-helices could act as sensors that modulate the flexibility of the functional loops via oligomerisation or protein-protein interactions, as the loops’ motions correlate well with the α-helices. The difference in the size of the β-barrel core and the α-helical bundle could be a factor that influences the separation of their mobility, and requires further investigation.

The rigidity of the β-barrel core is consistent with findings by others [42, 43] that it acts as a site of stabilising residues important for the integrity of the fold. For example, using a combination of computational approaches to define residue-residue contacts, hydrophobicity and amino acid conservation in 71 TBF structures, Gromiha *et al*. [43] found that the majority of the stabilising residues found in the β-barrel core have very low normalised B-factors, indicating a preference for immobility. The immobility of the β-barrel core that they describe mirrors our finding that the β-barrel core is very rigid (Figs 3 and 4).

We would also like to highlight the finding that oligomerisation acts to modulate the flexibility of the loop without changing the rigidity of the β-barrel core and the slightly more mobile α-helices (Fig 4). The observation from the fluctuations profile of the biological assemblies is consistent with the general flexibility patterns we have found in the analysis of the SSE flexibility of single TBF domains from each enzyme.

### The β-strands support long-distance correlations

The loop regions (including accessory secondary structures) are strongly correlated parts of TBF, with strong correlations running along the helices. The significant correlations between the β-strands in the core are fewer and within a shorter distance range, in contrast. Correlations that are implicated with the strands connect further to hubs of correlations associated with the loops and helices. The strands and helices are mostly correlated in motion via the loops at the N-terminal and C-terminal ends of the SSEs, and stronger correlations tend to occur within the accessory structures rather than the TIM barrel domain structure of the enzymes. This still holds for TBFs in their biological assembly form, yet the correlations in the regions away from the oligomeric interface are generally intensified (Cf. S9 Fig). Thus, we conclude that variation within the loop regions based on functionality could impact the mobility of the α-helices as well, and not that of the β-strands; a property that needs to be considered when designing TBF-based enzymes.

When looking at the correlation objects that span from the β-strands specifically, we see that the significant, distant correlations we defined span up to more than one β-α unit. However the numbers differ from structure to structure. This could be due to the fact that the number of correlations sampled using the percentiles changes with the size of the structure and is not a feature of the βαβ module of the structures, which has been suggested as the elementary module of the TBF [13, 19, 44, 45].

The slow motions of loops have been shown to be functionally important in some enzymes [46–48]. Katebi *et al*. showed, via ENM analysis that loops 6 and 7 of the TIM enzyme move in a concerted fashion [23]. We believe that the rigidity of the β-strands allows for distant correlations that are functionally relevant (cf. Fig 8 and S6 Fig), as demonstrated in other studies [5, 49].

In addition, Yang *et al*. [45] reported the presence of critical inter-SSE hydrogen-bonds between the main chain amino groups of odd-numbered β-strands and the side chain acceptors of the loops leading to their following α-helices, situated at the N-terminal end of the TIM enzyme structure. They believed that this was key to the stability of the fold and evidence for the modular evolution of the fold. Our ENM modelling does not take such interactions into account, as we only consider the Cα atoms of the proteins, but they are consistent with the correlations we observe between the β-strands and the α-helices via the loop regions that would reinforce this pattern.

### Catalytic residues lie in the rigid parts

The catalytic residues of all the structures analysed were found to be in the more rigid regions of the structure, whether they were found on the secondary structures or the loops, in terms of the normalised fluctuations. The catalytic residue positions were also found to have intermediate levels of deformation energy, consistent with studies which show active sites to be at the boundary of more deformable regions that allow appropriate conformational changes for substrate-binding [50, 51]. This is consistent with the idea that the positioning of catalytic residues in enzymes are conserved throughout evolution, as they are selected for optimal access to the substrate they act upon [52]. Katebi *et al*. also observed this trend in the TIM enzyme, where they concluded that the stability of the loops were important for catalysis [23]. In a large-scale computational analysis of 760 structures of enzymes belong to different folds, Chien & Huang defined the rigidity of the proteins using the weighted-contact network model and found that a significant proportion of catalytic residues lie in rigid environments [53]. We found that the phosphate and metal-binding sites also follow this trend, with the substrate binding sites to a lesser extent, depending on the superfamily.

The binding of the substrate is the biggest distinguishing feature between these structures, as in the case of the chitinase, where this is aided by the addition of a domain at the C-terminal end of the TBF. The TBF provides a number of rigid positions for the placement of catalytic residues, in particular at the C-terminal end of each β-strand that are geometrically close by the virtue of being symmetrical. Thus, nature can exploit a myriad of positions when placing an active site on the TBF. In fact, TBF has been referred to as a fold that is easily parameterisable for *de novo* protein design [54]. As a principle, it has been demonstrated that the rigidity of the peptide backbone of certain folds, such as the β-propeller, confers it a greater advantage for the grafting of new active sites than others that are deemed more flexible, due to the need for binding pocket complementarity for catalysis [55]. Thus, the success of the TBF as a scaffold for both natural and novel artificial enzymes cannot be decoupled from its intrinsic dynamic properties.

### Conclusion

We were able to find signature properties of the TIM barrel fold that are consistent regardless of sequence or functional conservation; i) the relative immobility of the inner β-barrel core between all the structures ii) the relative flexibility of the flanking α-helices ii) the strong, long-distance correlations of the strands in the immobile β-barrel core to the α-helices via the loops linking them. We determined that the preferred displacement types of the flanking α-helices consist of vertical and horizontal motions. We saw that this fold is successful in providing rigid positions for all catalytic residues, and that most other residue positions involved in function also share this property. We believe that the number of rigid positions offered by the TBF combined to the possibility of adding neighbouring loops or other accessory elements is key to explaining its versatility. Our results also show that comparative flexibility studies are highly sensitive to the alignments used.

It is really striking that the main patterns of the intrinsic dynamics in the TBF are as conservative as the fold, and not dependent on function. This result is supported by the findings of Zen *et al*.[9] who reported that flexibility measures could be used to satisfactorily align TIM barrels with different functions as captured by their EC classification. It is also remarkable that signature flexibility pattern is present independent of the oligomerisation state, which otherwise tends to affect the loop and α-helical regions. We believe that this conservative intrinsic dynamics of the TIM barrel scaffold further characterises its success as a versatile platform for many types of enzymatic reactions. We find that despite the varying levels of structural and sequence homology, the overall shape is able to determine global similarities in intrinsic dynamics. We further suggest that sequence or functional similarity may not be the main driving force in the conservation of intrinsic dynamics. The characterisation of the flexibility of the TBF also has implications in protein and drug design, in that by exploiting the intrinsic dynamic signatures, one could provide solutions that were previously limited to specific target areas.

## Methods

### Dataset preparation

We used protein structures from five different superfamilies, referred to as Triose phosphate isomerase (TIM), Aldolase class I (ALD1), Enolase (ENOL), Chitinase (CHTN) subfamily of the Glycosidases (GLYC) and Phosphatidylinositol (PI) phospholipase C (PIPLC) in Nagano *et al*. [10], summarised in S1 Table. According to the phylogenetic analysis in this review, the protein families (nomenclature used is in parentheses) cluster according to the following groupings where TIM and ALD1 are closely linked, followed by ENOL, with CHTN and PIPLC being distant outliers. CHTN and PIPLC are also considered to be two of the four superfamilies to have little evolutionary link to the rest of the superfamilies. These superfamilies relate to each other at the Topology level in the CATH database [56] as of January 2011. Since then, two of the superfamilies, TIM and ALD1, have been reclassified to be part of the same Homology level [57].

For the purposes of comparison, we picked five representative structures from each of these superfamilies, which are further annotated in (S2 Table). These structures all exist as part of dimers, and have varying lengths that include additional secondary structures, further illustrated in S1 Fig. The structures are also treated as monomers, even though most come as dimers. As the enzymes chosen are subject to the CATH domain classification, we found that it is appropriate to exploit the structural information that the classification provides as a starting point, as has been done previously by Zen *et al*. [9]. Moreover, we find the conformation of a subunit isolated from an oligomer is able to capture the influence of the interactions of other subunits [58]. The structures were prepared according to the domain annotation found in CATH, which included the truncation of 2 structures: the first domain of 1KKO and a domain sitting on loop 7 of 1E15. This resulted in a set of structures with varying length, with 1KKO as the smallest at 246 amino acids, followed closely by 1N55 (248), then 3CH0 (271), 3CWN (315) and 1E15 (355). Three of the five structures bind to a phosphate moiety in their substrate (1N55, 3CWN and 3CH0), while two structures, 1KKO and 3CH0, have Mg^2+^ and Ca^2+^ metal ions as co-factors respectively (S1 Table). There is no consensus on the positions of their catalytic or substrate binding sites on the fold. We also investigated the biological assemblies as provided by the Protein Data Bank (PDB) for comparison (Cf S3 Table).

For the first half of the analysis, we also include homologues from each of these superfamilies. The homologues were retrieved from Blastp, searched against the PDB database. The lowest E-values were chosen for each, where the hits were not identical to the query sequence (i.e. below 99% identical) and did not have the same taxonomic rank. This led us to pick four additional structures for all the superfamilies except for the Enolase, where only two other structures were found to fit the criteria, resulting in a total of 23 structures analysed (S2 Table).

### Sequence and structural alignment

The sequence alignments performed to determine sequence identities between the various structures were generated using MUSCLE [59] as implemented in JALVIEW [60]. The web service SIAS (http://imed.med.ucm.es/Tools/sias.html) was then used to calculate the sequence identity which is defined as the following:

$$S=100\cdot (\frac{I}{L})$$ (1)

where *I* is the number of identical residues and *L* is the length of the alignment, including the gaps.

We obtained the structural alignments from MUSTANG [36]. This alignment program was one of the top three performers in a benchmarking study [61] and at aligning TIM Barrel proteins reliably [25]. It aligns the structures using the topological information from the Cα atoms in the backbone via an optimised progressive pairwise algorithm. The resulting alignment was used in the FASTA format for the comparative analysis of the intrinsic dynamics.

### Elastic Normal Mode calculations

To obtain the description of the intrinsic dynamics of these structures, we first constructed their Elastic Network Model (ENM) for normal mode analysis (NMA). The ENMs were constructed using the Cα force field[62], as implemented in Molecular Modelling Toolkit [63]. Each amino acid is represented by a mass at the position of its Cα atom. The following pair potential describes the interaction between two Cα atoms,

$$V_{ij}\left(r\right)=\frac{k_{ij}}{2}{\left(\|r_{ij}\|-\|r_{ij}^{0}\|\right)}^{2}$$ (2)

where:

$$k_{ij}=\left\{ \begin{array}{l} a\;r_{ij}^{0}-b,\;\text{for}\;r_{ij}^{0}<d\\ c{\left(r_{ij}^{0}\right)}^{-6},\;\text{for}\;r_{ij}^{0}\geq d \end{array}\right.$$ (3)

**r**_*ij*_ is the pair distance vector between two Cα atom positions *i* and *j*, while **r**_*ij*_^0^ is the corresponding pair distance vector in the input configuration. Hinsen [62] parameterised the force constants *k*_*ij*_ in the construction of the force field, such that *a * =  8.6x105 kJ mol^−1^ nm^−3^; *b*  =  2.39x105 kJ mol^−1^ nm^2^; *c*  =  128 kJ mol^−1^ nm^4^ and *d * =  0.4 nm.

The potential energy of a configuration **r** of the ENM is then:

$$V(r)=\sum_{i=1}^{N}\sum_{j=i+1}^{N}V_{ij}(r)$$ (4)

The normal modes are eigenvectors of the mass weighted matrix of second order partial derivatives of the potential *V*. The eigenvalues correspond to the squares of the frequencies for each mode.

### Bhattacharyya coefficient score

The Bhattacharyya coefficient (BC) score is calculated based on Fuglebakk [37], as it is implemented in WEBnm@ [64]. The BC score compares the effective covariances, from the subset modes of the aligned cores of two structures, (*A* and *B*), such that:

$$\text{BC}=\text{exp}\;\left(-\frac{1}{2}\text{ln}\;\left[|\frac{1}{2}\;\left(\tilde{A}+\tilde{B}\right)|\;{\left(|\tilde{A}||\tilde{B}|\right)}^{-\frac{1}{2}}\right]\right)$$ (5)

Here |**X**| denotes the determinant of **X** and the rank of the matrices are reduced in two steps: First, **A**_*n*_ and **B**_*m*_ are obtained from the *n* and *m* lowest frequency modes of their respective proteins and normalised by dividing by their trace. Then, $\tilde{A}$ and $\tilde{B}$ are obtained by projecting **A**_*n*_ and **B**_*m*_ on to *s* eigenvectors of (**A**_*n*_+**B**_*m*_)/2 that explain most of its variance. For each comparison *n* and *m* are chosen so that 95% of the variance of each protein is retained and *s* so that 75% of the variance of (**A**_*n*_+**B**_*m*_)/2 is retained.

### Normalised fluctuations

The fluctuation of the Cα atom of each residue can be described as a sum over its displacement for all non-trivial modes that are weighted by their eigenvalues. The fluctuation for each residue position, *F*_*i*_ is given by the equation:

$$F_{i}=\sum_{m=1}^{3N-6}\frac{{\left||{\left[d_{m}\right]}_{i}|\right|}^{2}}{\lambda_{m}}$$ (6)

where *λj* is the eigenvalue of mode *j*, *N* is the number of modes, and [**d**_*m*_]_*i*_ is the displacement vector for residue *i* in mode *m*.

### Normalised deformations

The normalised deformations are based on the definition in [65]. The deformation energy for each Cα atom, for a single mode is given by:

$$E_{i}=\frac{N}{\sum_{j=1}^{N}{\left|d_{j}\right|}^{2}}\cdot \frac{1}{2}\sum_{j=1}^{N}\frac{k_{ij}{\left|\left(d_{i}-d_{j}\right)\cdot \left(r_{i}^{0}-r_{j}^{0}\right)\right|}^{2}}{{\left|r_{i}^{0}-r_{j}^{0}\right|}^{2}}$$ (7)

where *N* is the number of Cα atoms in the protein, **d**_*i*_ and **d**_*j*_ are the displacement vectors of atoms *i* and *j* and **r**_*i*_^0^-**r**_*j*_^0^ is the corresponding pair distance vector in the input configuration, respectively. The normalised deformation energy (*D*_*i*_) for a particular Cα atom position *i* is summed and then normalised over all non-trivial modes (3*N*-6) by the following:

$$D_{i}\;=\;\frac{\sum_{m=1}^{3N-6}{\left[E_{m}\right]}_{i}}{3N-6}$$ (8)

where [*E*_*m*_]_*i*_ is the deformation energy *E*_*i*_ from (7) for mode *m*.

### Significant correlations

The correlation matrix as defined by Ichiye and Karplus [66] is calculated from the normal modes. Each element in the matrix quantifies the coupling between two atoms *i* and *j* as:

$$C_{ij}\;=\;\frac{\sum_{m=1}^{3N-6}\frac{1}{\lambda_{m}}{\left[v_{m}\right]}_{i}\cdot {\left[v_{m}\right]}_{j}}{{(\sum_{m=1}^{3N-6}{\left[v_{m}\right]}_{i}\cdot {\left[v_{m}\right]}_{i})}^{\frac{1}{2}}\cdot {(\sum_{m=1}^{3N-6}{\left[v_{m}\right]}_{j}\cdot {\left[v_{m}\right]}_{j})}^{\frac{1}{2}}}$$ (9)

where **v**_*m*_ and *λ*_*m*_ are eigenvectors and eigenvalues of the *m*^th^ normal mode respectively and the *i* and *j* subscripts denote the component of the mode corresponding to individual atoms, summed over all non-trivial modes. *C*_*ij*_ is the expected inner product of displacements of atom *i* and *j*, and ranges from –1 to 1, where –1 and 1 are maximal anti-correlations and correlations, respectively, and 0 represents a lack of any correlation.

For visual inspection, strong *C*_*ij*_ correlation scores in the correlation matrix collected as objects are represented as sticks in Figs 6 and 8, as implemented in [39]. The correlation scores are chosen to reflect the 95^th^ percentile rank of their absolute values, because their magnitudes describe correlations of the same strength. We chose a percentile threshold instead of a threshold based on the value of the pairwise correlation because of its strength to identify the most significant correlations in a protein structure. In the absence of such a criteria, the choice of a threshold based on a hard correlation value cut-off, would imply that we arbitrarily decide which correlation values are relevant without a reliable reference. The correlated pairs of Cα atoms are later separated by positions that have positive correlations above the 95^th^ percentile and those that have negative correlations below the negative of this score. Furthermore, only the correlations between atoms that are at least 8Å apart are considered, to filter out the pairs of Cα atoms whose correlations are along the peptide backbone and are heavily influenced by adjacent bonding and interactions due to close proximity. These pairs of Cα atoms are also linked by the springs with the stronger force constants in the ENM. The distance threshold is reduced to 4Å, while the score threshold is increased to the 97.5^th^ percentile rank when examining signification correlations that originate at the β-strands, as it corresponds to the approximate distance of the Cα atoms in adjacent strands (S8 Fig). The objects resulting from the search are visualised using the molecular graphics program PyMOL [67] as sticks between atom pairs, in red when positive and blue when negative.

### Overlap between normalised ideal vector displacements of secondary structure elements and ENM modes

The ideal vector overlap is a method that allows the characterisation of the normal modes vectors as simplified displacements in the protein structure [40]. The calculation of the dot product (overlap) between a displacement vector and the full set of normal modes identifies which modes contribute most to the given displacement. Low energy modes are characterised by larger amplitude motions while higher energy modes describe motions with smaller amplitudes. Hence displacements contributed by low energy modes will have larger amplitudes than those contributed to by higher energy modes. We defined two types of displacement vectors: i) normalised rotational and vertical displacements of the α-helical bundles and β-barrel cores (8 SSEs each) of the five main TBF structures analysed (Fig 11A and S1 Methods), ii) vectors describing vertical, horizontal, tilting and bending displacements of the N- and C-terminal halves of individual SSEs (Fig 11B and S1 Methods).

The overlap score, Ω_*w*,_ to evaluate these is given by:

$$\Omega_{w}\;=\;\sum_{m=1}^{3N-6}\;\lambda_{m}{\left(z\cdot v_{m}\right)}^{2}$$ (10)

where **v**_*m*_ is the normal mode vector of mode *m*, and **z** is the 3*N* normalised ideal vector (as defined in S1 Methods), where *N* is the number of Cα atoms. The Ω_*w*_ score is a cumulative sum that includes the energetic contribution, such that the sum of the squared overlaps is weighted by the modes’ eigenvalue over all non-trivial modes. This results in a score which is high if the displacement is energetically unfavourable, and vice-versa.

## Supporting Information

S1 Fig Scheme of the two-dimensional SSE arrangement as viewed from the top of the C-terminal end of each enzyme, identified by PDB ID. The diversification of the fold occurs with the addition of secondary structure elements, typically at the C-terminal end (smaller circles and triangles). The triangles represent the β-strands, the circles represent the α-helices, while the blue and yellow squares are the N- and C-termini respectively. The black dotted lines represent the circular spatial arrangement of the TBF, such that the β-stands are able to close and form a parallel β-barrel core, flanked by their α-helices. Red denotes the position of the catalytic residues, while cyan denotes the site of substrate-binding. The shapes are filled if the key residues are positioned on the SSE and outlined if they are positioned on the loop between two SSEs. The green stars refer to the position of metal ion-binding sites, while the gold stars refer to phosphate-binding sites. The red dotted line in 1E15 refers to the truncation of a domain that leaves behind two free ends at the loop region between the 7th β-strand and its flanking α-helix.
(PDF)

S2 Fig Sequence representation of the MUSTANG structure alignment of the main five TBF structures. The α-helices and β-strands are indicated in purple and dark teal, respectively. Red boxes denote parts of the alignment that are conserved between all five sequences.
(PDF)

S3 Fig Sequence identity based on the MUSCLE alignment between the five main TBF proteins from different superfamilies (left) and with the addition of homologous TBF proteins from each of these superfamilies (right). All of these proteins are identified by their PDB IDs. The colour scale goes from blue to yellow to red, for low (20%) to high (100%) sequence identity percentages. The dendrogram reflects the hierarchical clustering of the structures based on their sequence identity.
(PDF)

S4 Fig Normalised fluctuations of the five TIM superfamilies and their orthologues. Green bars show α-helical regions, while red show the β-stranded regions. The sixth panel (bottom right) is a zoomed in profile of 1N55.
(PDF)

S5 Fig Normalised deformation energies (calculated over all non-trivial normal modes). Red shows high deformation energies, while blue shows low energies, with white as the intermediate values. Scale ranges from low (blue) to intermediate (white) to high (red) normalised values of the deformation energies.
(PDF)

S6 Fig Distribution of distant significant correlations in the five TBF structures. On the left side of the image, we have the top views from the perspective of the C-terminal end, where the respective structures are displayed with the cartoon representation in green, and sticks between each pair of residue positions with significant correlations at least 8 Å apart (cf. Methods). The red sticks indicate positive correlations above the score threshold at the 95th percentile rank of the absolute values of the correlations. The blue sticks indicate negative values of correlations below the negative of the score threshold. The yellow spheres represent the positions of the catalytic amino acids while the purple, cyan and orange spheres represent substrate, phosphate and metal-ion binding residues respectively. On the right, we have a side-profile, clipped view of the TBF, with the N-terminal end at the bottom, and the C-terminal end at the top.
(PDF)

S7 Fig Correlation heatmaps of the main five TBF structures. Dotted black lines refer to the boundaries of the β-strands, whereas the green dotted lines indicate the helices in between them. The red and blue pixels indicate positive and negative correlations respectively.
(PDF)

S8 Fig Significant short-range correlations involving β-strands in the five TBF structures. The Cα atoms of catalytic residues are represented as yellow spheres, the substrate binding by purple spheres, the phosphate binding by cyan spheres, and the metal ion binding by orange spheres. On the left side, there are the top views from the C-terminal end of the structures (green), where the red sticks signify short-range (at least 4 Å apart) above the 95^th^ percentile rank of the absolute correlations. On the right, we have the short-range correlations with the scores above the 97.5^th^ percentile rank of the absolute correlations. In both cases, we see that the β-strands prefer to mediate strong correlations at close range with each other.
(PDF)

S9 Fig **Distribution of distant significant distant correlations in the monomeric form (A) and chain A of the dimeric form (B) of 1N55.** We have the top views from the perspective of the C-terminal end, where the respective structures are displayed with the cartoon representation in rainbow (N-terminal in blue, C-terminal in red), and sticks between each pair of residue positions with significant correlations at least 8 Å apart (cf. Methods). The red sticks indicate positive correlations above the score threshold at the 95^th^ percentile rank of the absolute values of the correlations. The oligomeric interface spans the first three β-α secondary structure units (blue to cyan).
(PDF)

S1 Table Summary of the five main TBF structures studied. (PDF)

S2 Table TBF structures and their additional homologues. (PDF)

S3 Table Oligomeric forms of the TBF enzyme dataset, as defined by the PDB and PISA. (PDF)

S1 Methods Definition of the normalised ideal vectors. (PDF)
