# Supplementary material for: Similarity in Shape Dictates Signature Intrinsic Dynamics Despite No Functional Conservation in TIM Barrel Enzymes
Source: PLoS Comput Biol. 2016 Mar 25;12(3):e1004834. doi: 10.1371/journal.pcbi.1004834 (PMC4807811; doi:10.1371/journal.pcbi.1004834)
Supplement: S1 Fig — The diversification of the fold occurs with the addition of secondary structure elements, typically at the C-terminal end (smaller circles and triangles). The triangles represent the β-strands, the circles represent the α-helices, while the blue and yellow squares are the N- and C-termini respectively. The black dotted lines represent the circular spatial arrangement of the TBF, such that the β-stands are able to close and form a parallel β-barrel core, flanked by their α-helices. Red denotes the position of the catalytic residues, while cyan denotes the site of substrate-binding. The shapes are filled if the key residues are positioned on the SSE and outlined if they are positioned on the loop between two SSEs. The green stars refer to the position of metal ion-binding sites, while the gold stars refer to phosphate-binding sites. The red dotted line in 1E15 refers to the truncation of a domain that leaves behind two free ends at the loop region between the 7th β-strand and its flanking α-helix. (PDF) [file pcbi.1004834.s001.pdf]

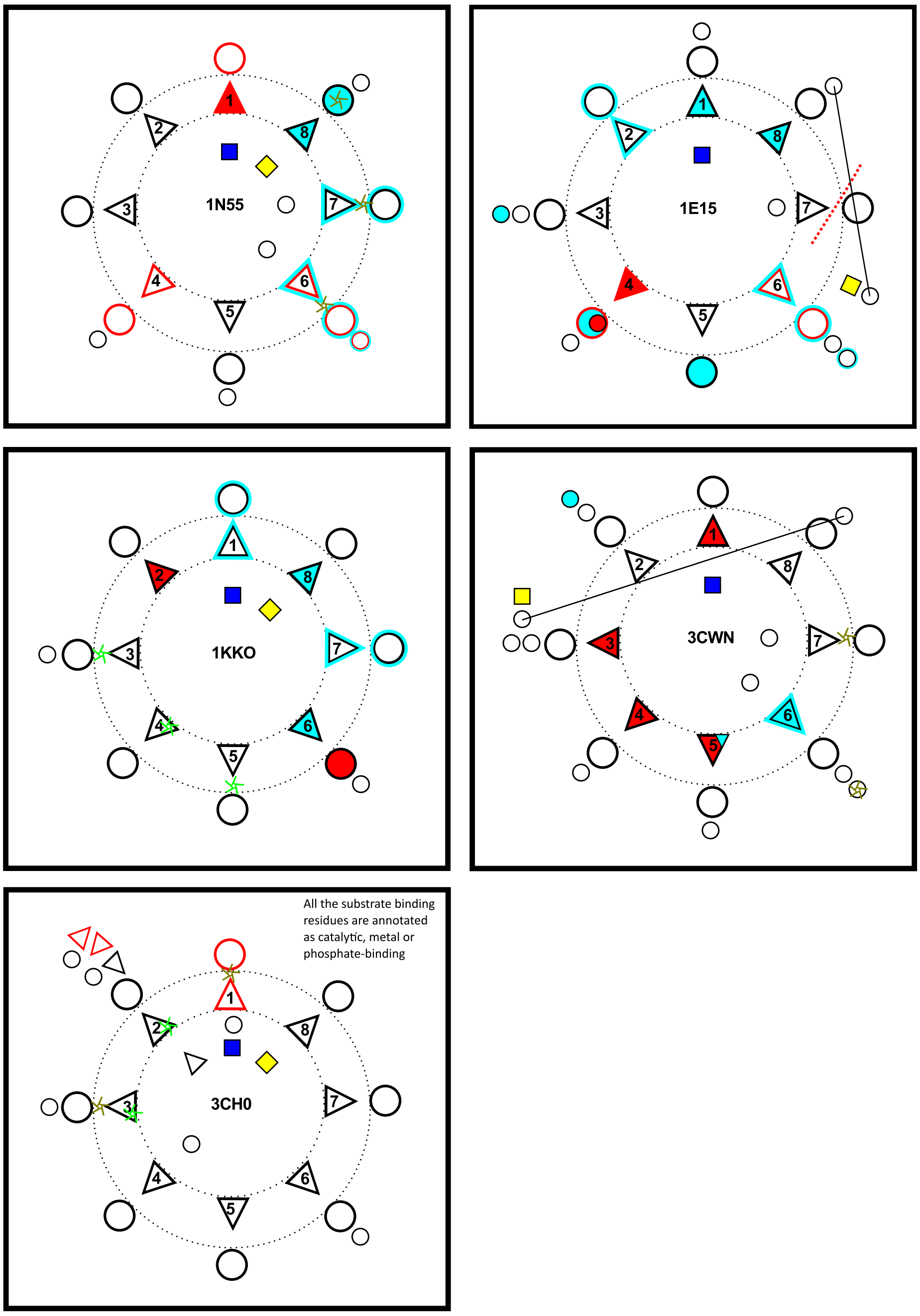

Supplementary Figure 1 – Scheme of the two-dimensional SSE arrangement as viewed from the top of the C-terminal end of each enzyme, identified by PDB ID. The diversification of the fold occurs with the addition of secondary structure elements, typically at the C-terminal end (smaller circles and triangles). The triangles represent the β-strands, the circles represent the α-helices, while the blue and yellow squares are the N- and C-termini respectively. The black dotted lines represent the circular spatial arrangement of the TBF, such that the β-stands are able to close and form a parallel β-barrel core, flanked by their α-helices. Red denotes the position of the catalytic residues, while cyan denotes the site of substrate-binding. The shapes are filled if the key residues are positioned on the SSE and outlined if they are positioned on the loop between two SSEs. The green stars refer to the position of metal ion-binding sites, while the gold stars refer to phosphate-binding sites. The red dotted line in 1E15 refers to the truncation of a domain that leaves behind two free ends at the loop region between the 7th β-strand and its flanking α-helix.
