# Supplementary material for: Similarity in Shape Dictates Signature Intrinsic Dynamics Despite No Functional Conservation in TIM Barrel Enzymes
Source: PLoS Comput Biol. 2016 Mar 25;12(3):e1004834. doi: 10.1371/journal.pcbi.1004834 (PMC4807811; doi:10.1371/journal.pcbi.1004834)
Supplement: S2 Fig — The α-helices and β-strands are indicated in purple and dark teal, respectively. Red boxes denote parts of the alignment that are conserved between all five sequences. (PDF) [file pcbi.1004834.s002.pdf]

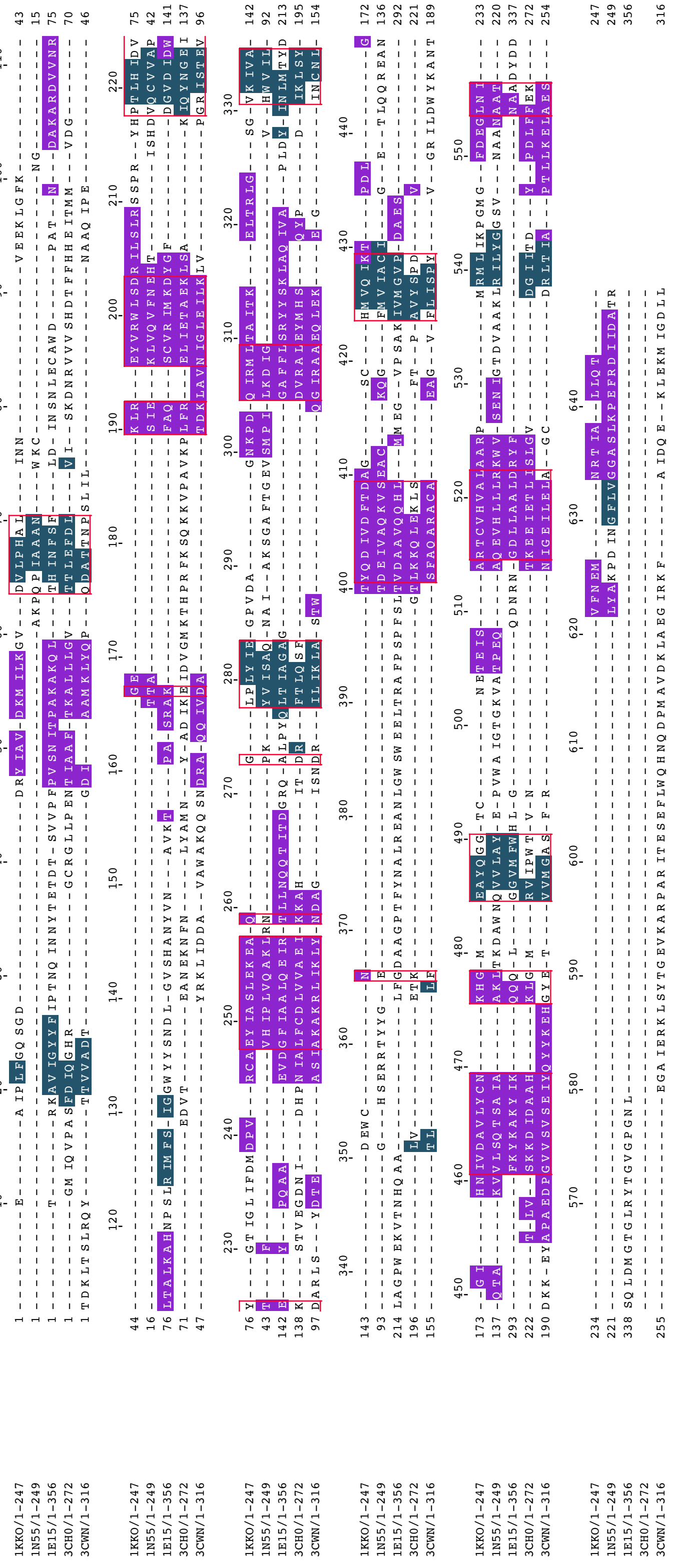

Supplementary Figure 2 – Sequence representation of the MUSTANG structure alignment of the main five TBF structures. The α-helices and β-strands are indicated in purple and dark teal, respectively. Red boxes denote parts of the alignment that are conserved between all five sequences.
