# Supplementary material for: Similarity in Shape Dictates Signature Intrinsic Dynamics Despite No Functional Conservation in TIM Barrel Enzymes
Source: PLoS Comput Biol. 2016 Mar 25;12(3):e1004834. doi: 10.1371/journal.pcbi.1004834 (PMC4807811; doi:10.1371/journal.pcbi.1004834)
Supplement: S3 Fig — All of these proteins are identified by their PDB IDs. The colour scale goes from blue to yellow to red, for low (20%) to high (100%) sequence identity percentages. The dendrogram reflects the hierarchical clustering of the structures based on their sequence identity. (PDF) [file pcbi.1004834.s003.pdf]

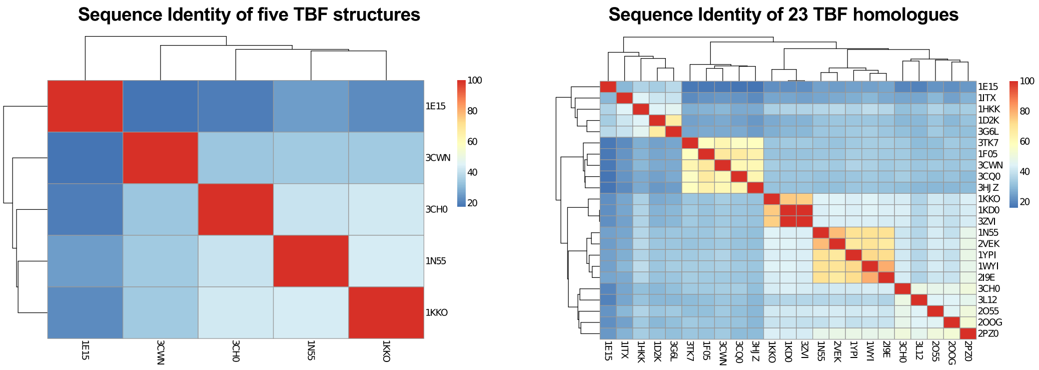

Supplementary Figure 3 – Sequence identity based on the MUSCLE alignment between the five main TBF proteins from different superfamilies (left) and with the addition of homologous TBF proteins from each of these superfamilies (right). All of these proteins are identified by their PDB IDs. The colour scale goes from blue to yellow to red, for low (20%) to high (100%) sequence identity percentages. The dendrogram reflects the hierarchical clustering of the structures based on their sequence identity.
