# Supplementary material for: Similarity in Shape Dictates Signature Intrinsic Dynamics Despite No Functional Conservation in TIM Barrel Enzymes
Source: PLoS Comput Biol. 2016 Mar 25;12(3):e1004834. doi: 10.1371/journal.pcbi.1004834 (PMC4807811; doi:10.1371/journal.pcbi.1004834)
Supplement: S4 Fig — Green bars show α-helical regions, while red show the β-stranded regions. The sixth panel (bottom right) is a zoomed in profile of 1N55. (PDF) [file pcbi.1004834.s004.pdf]

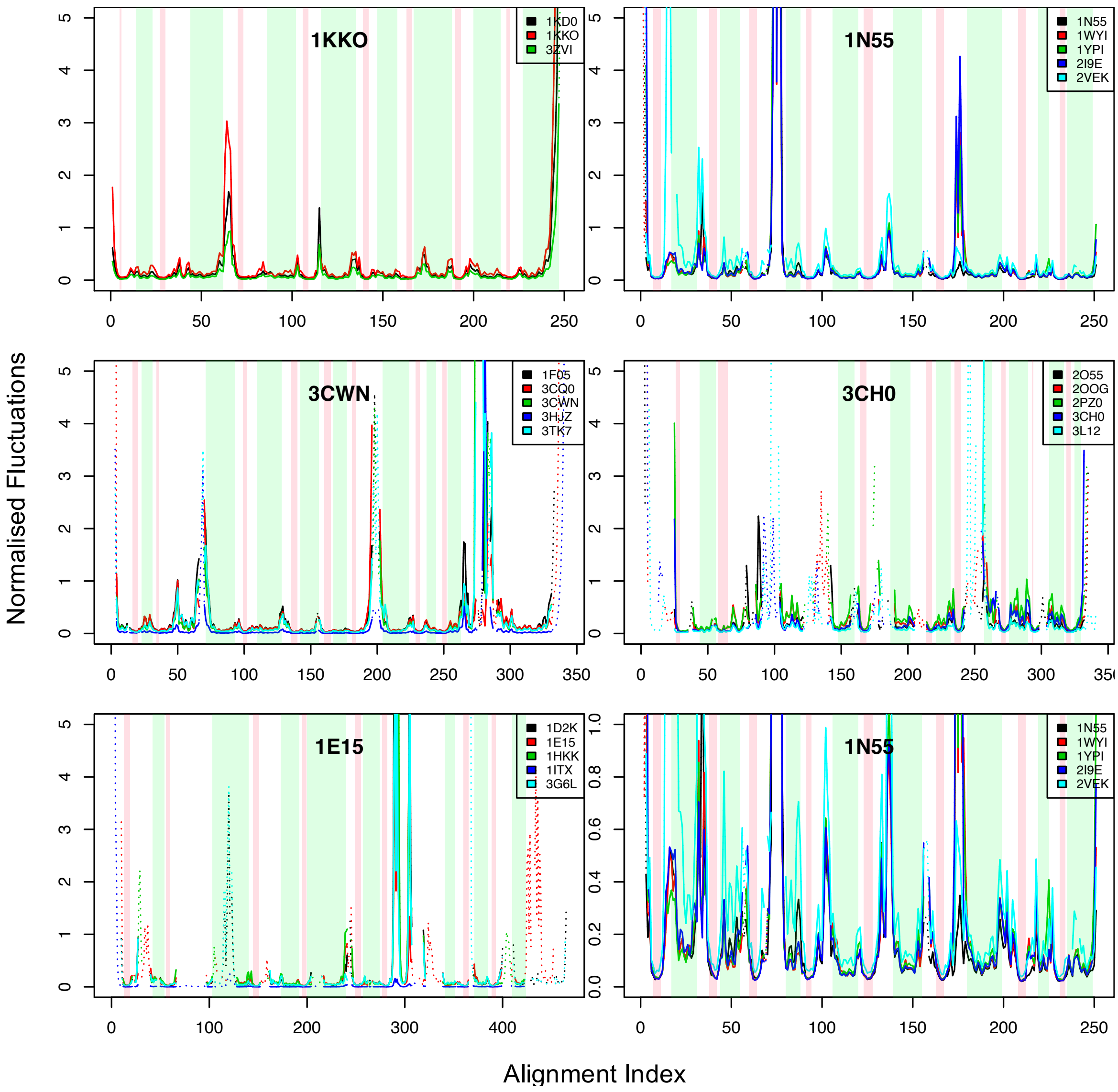

Supplementary Figure 4 – Normalised fluctuations of the five TIM superfamilies and their orthologues. Green bars show α-helical regions, while red show the β-stranded regions. The sixth panel (bottom right) is a zoomed in profile of 1N55.
