# Supplementary material for: Similarity in Shape Dictates Signature Intrinsic Dynamics Despite No Functional Conservation in TIM Barrel Enzymes
Source: PLoS Comput Biol. 2016 Mar 25;12(3):e1004834. doi: 10.1371/journal.pcbi.1004834 (PMC4807811; doi:10.1371/journal.pcbi.1004834)
Supplement: S5 Fig — Red shows high deformation energies, while blue shows low energies, with white as the intermediate values. Scale ranges from low (blue) to intermediate (white) to high (red) normalised values of the deformation energies. (PDF) [file pcbi.1004834.s005.pdf]

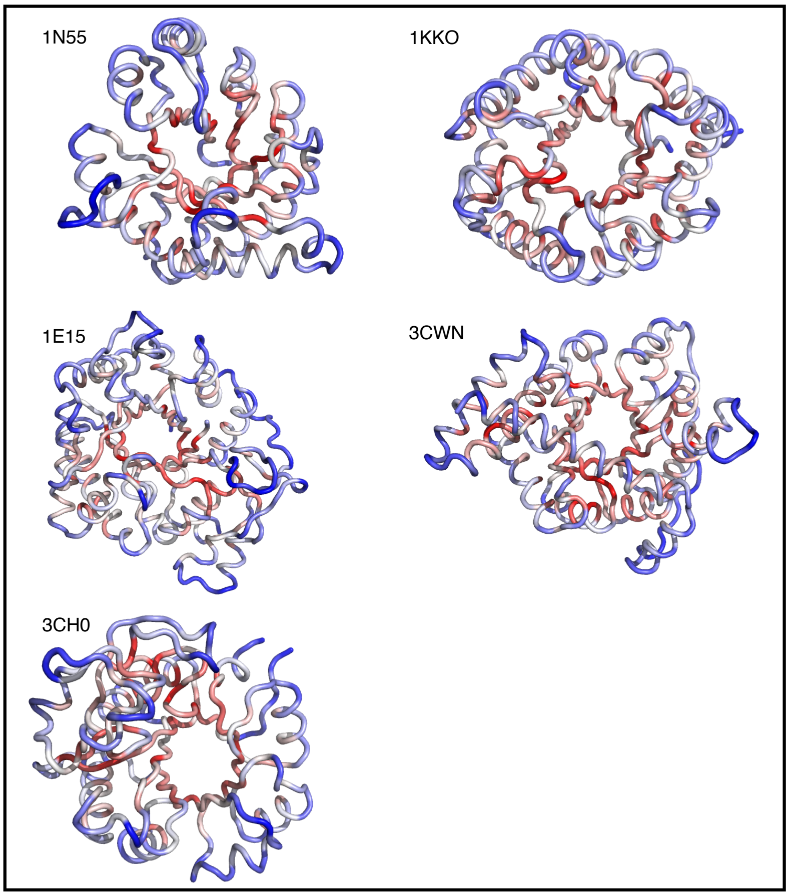

Supplementary Figure 5 – Normalised deformation energies (calculated over all non-trivial normal modes). Red shows high deformation energies, while blue shows low energies, with white as the intermediate values. Scale ranges from low (blue) to intermediate (white) to high (red) normalised values of the deformation energies.
