# Supplementary material for: Similarity in Shape Dictates Signature Intrinsic Dynamics Despite No Functional Conservation in TIM Barrel Enzymes
Source: PLoS Comput Biol. 2016 Mar 25;12(3):e1004834. doi: 10.1371/journal.pcbi.1004834 (PMC4807811; doi:10.1371/journal.pcbi.1004834)
Supplement: S6 Fig — On the left side of the image, we have the top views from the perspective of the C-terminal end, where the respective structures are displayed with the cartoon representation in green, and sticks between each pair of residue positions with significant correlations at least 8 Å apart (cf. Methods). The red sticks indicate positive correlations above the score threshold at the 95th percentile rank of the absolute values of the correlations. The blue sticks indicate negative values of correlations below the negative of the score threshold. The yellow spheres represent the positions of the catalytic amino acids while the purple, cyan and orange spheres represent substrate, phosphate and metal-ion binding residues respectively. On the right, we have a side-profile, clipped view of the TBF, with the N-terminal end at the bottom, and the C-terminal end at the top. (PDF) [file pcbi.1004834.s006.pdf]

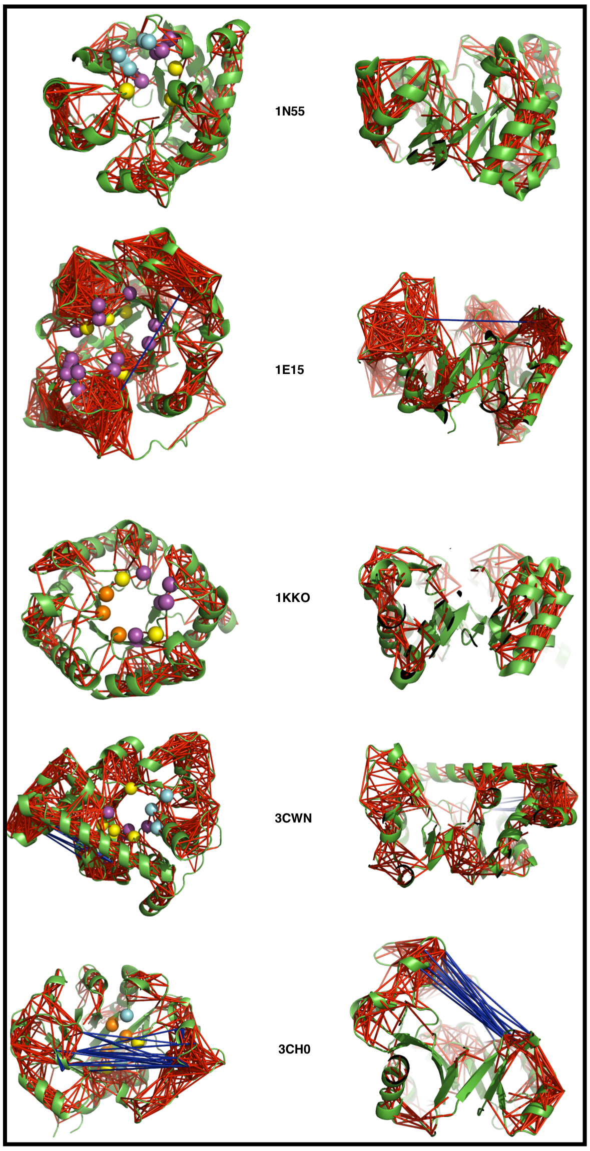

Supplementary Figure 6 - Distribution of distant significant correlations in the five TBF structures. On the left side of the image, we have the top views from the perspective of the C-terminal end, where the respective structures are displayed with the cartoon representation in green, and sticks between each pair of residue positions with significant correlations at least 8 Å apart (cf. Methods). The red sticks indicate positive correlations above the score threshold at the 95th percentile rank of the absolute values of the correlations. The blue sticks indicate negative values of correlations below the negative of the score threshold. The yellow spheres represent the positions of the catalytic amino acids while the purple, cyan and orange spheres represent substrate, phosphate and metal-ion binding residues respectively. On the right, we have a side-profile, clipped view of the TBF, with the N-terminal end at the bottom, and the C-terminal end at the top.
