# Supplementary material for: Similarity in Shape Dictates Signature Intrinsic Dynamics Despite No Functional Conservation in TIM Barrel Enzymes
Source: PLoS Comput Biol. 2016 Mar 25;12(3):e1004834. doi: 10.1371/journal.pcbi.1004834 (PMC4807811; doi:10.1371/journal.pcbi.1004834)
Supplement: S7 Fig — Dotted black lines refer to the boundaries of the β-strands, whereas the green dotted lines indicate the helices in between them. The red and blue pixels indicate positive and negative correlations respectively. (PDF) [file pcbi.1004834.s007.pdf]

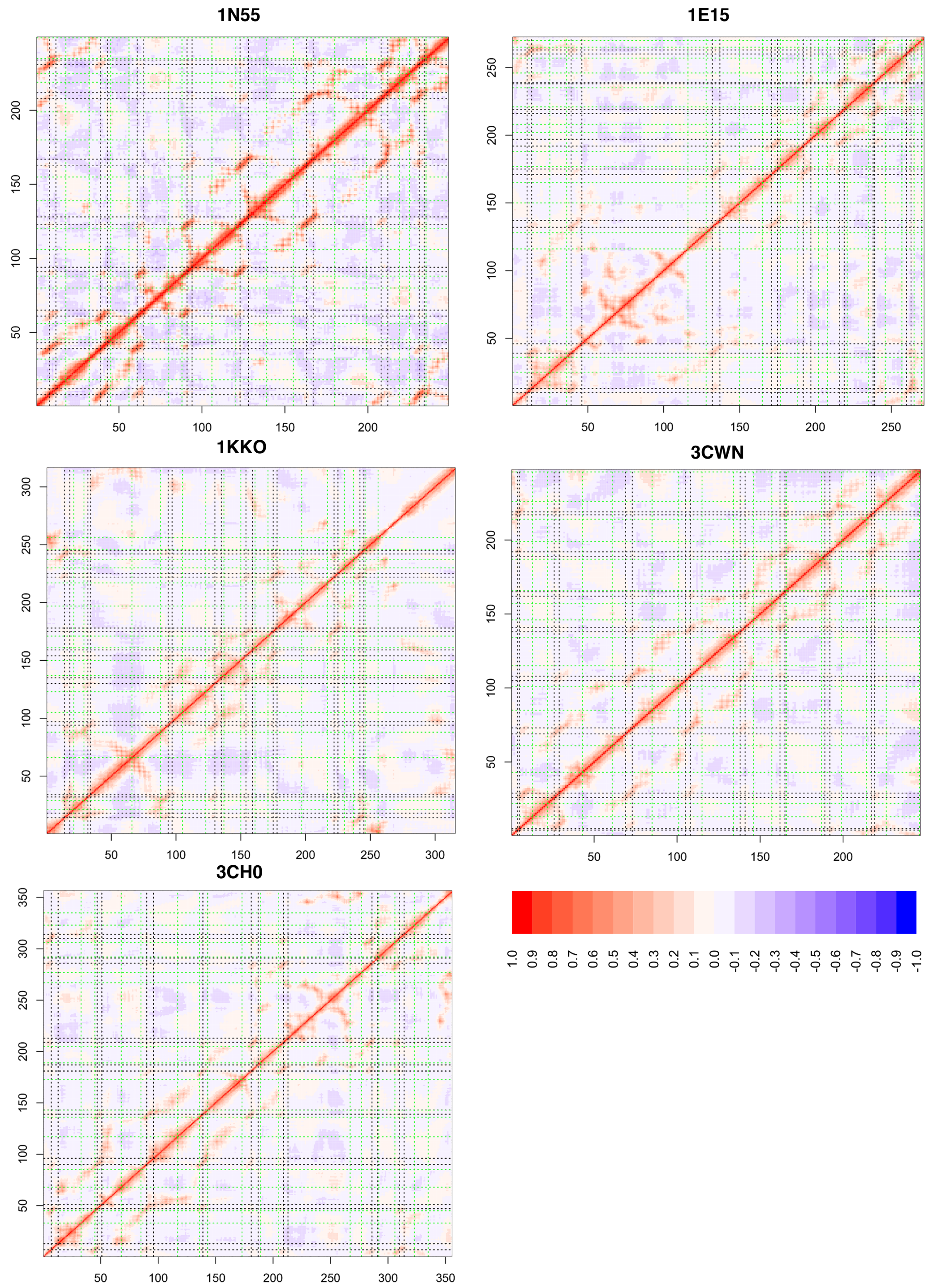

Supplementary Figure 7 – Correlation heatmaps of the main five TBF structures. Dotted black lines refer to the boundaries of the beta strands, whereas the green dotted lines indicate the helices in between them. The red and blue pixels indicate positive and negative correlations respectively.
