# Supplementary material for: Similarity in Shape Dictates Signature Intrinsic Dynamics Despite No Functional Conservation in TIM Barrel Enzymes
Source: PLoS Comput Biol. 2016 Mar 25;12(3):e1004834. doi: 10.1371/journal.pcbi.1004834 (PMC4807811; doi:10.1371/journal.pcbi.1004834)
Supplement: S8 Fig — The Cα atoms of catalytic residues are represented as yellow spheres, the substrate binding by purple spheres, the phosphate binding by cyan spheres, and the metal ion binding by orange spheres. On the left side, there are the top views from the C-terminal end of the structures (green), where the red sticks signify short-range (at least 4 Å apart) above the 95th percentile rank of the absolute correlations. On the right, we have the short-range correlations with the scores above the 97.5th percentile rank of the absolute correlations. In both cases, we see that the β-strands prefer to mediate strong correlations at close range with each other. (PDF) [file pcbi.1004834.s008.pdf]

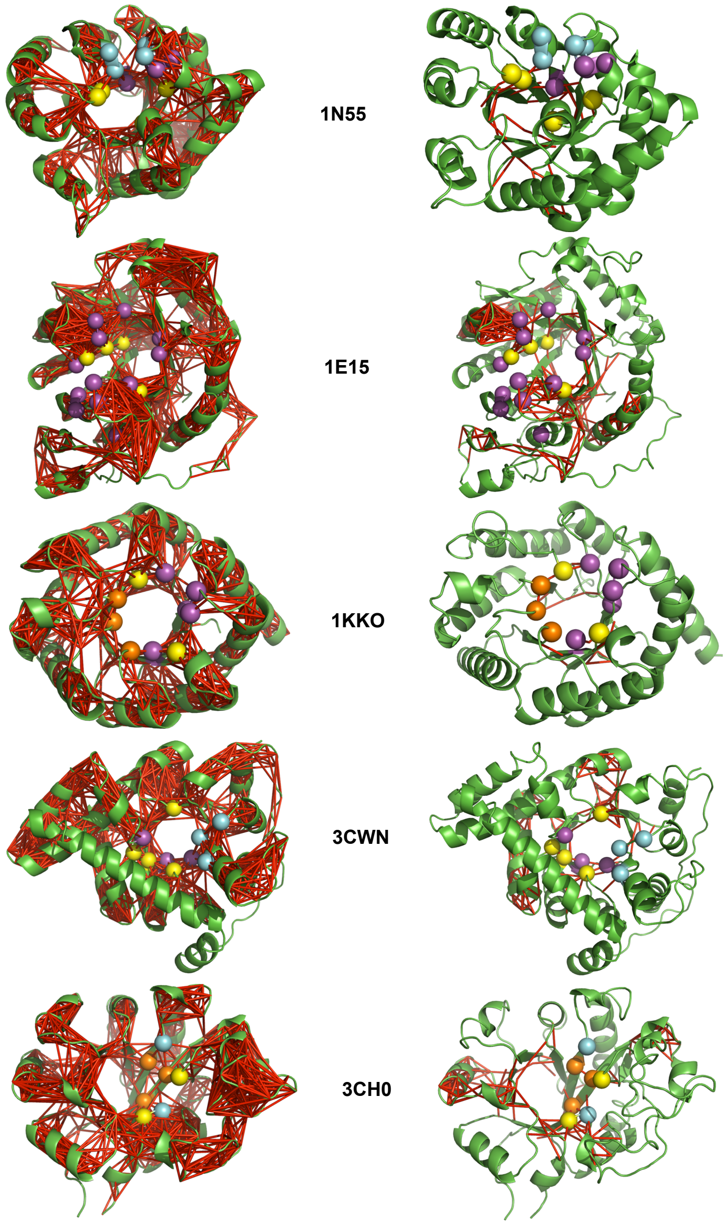

Supplementary Figure 8 - Significant short-range correlations involving β-strands in the five TBF structures. The Cα atoms of catalytic residues are represented as yellow spheres, the substrate binding by purple spheres, the phosphate binding by cyan spheres, and the metal ion binding by orange spheres. On the left side, there are the top views from the C-terminal end of the structures (green), where the red sticks signify short-range (at least 4 Å apart) above the 95th percentile rank of the absolute correlations. On the right, we have the short-range correlations with the scores above the 97.5^{th} percentile rank of the absolute correlations. In both cases, we see that the β-strands prefer to mediate strong correlations at close range with each other.
