# Supplementary material for: Similarity in Shape Dictates Signature Intrinsic Dynamics Despite No Functional Conservation in TIM Barrel Enzymes
Source: PLoS Comput Biol. 2016 Mar 25;12(3):e1004834. doi: 10.1371/journal.pcbi.1004834 (PMC4807811; doi:10.1371/journal.pcbi.1004834)
Supplement: S9 Fig — Distribution of distant significant distant correlations in the monomeric form (A) and chain A of the dimeric form (B) of 1N55. We have the top views from the perspective of the C-terminal end, where the respective structures are displayed with the cartoon representation in rainbow (N-terminal in blue, C-terminal in red), and sticks between each pair of residue positions with significant correlations at least 8 Å apart (cf. Methods). The red sticks indicate positive correlations above the score threshold at the 95th percentile rank of the absolute values of the correlations. The oligomeric interface spans the first three β-α secondary structure units (blue to cyan). (PDF) [file pcbi.1004834.s009.pdf]

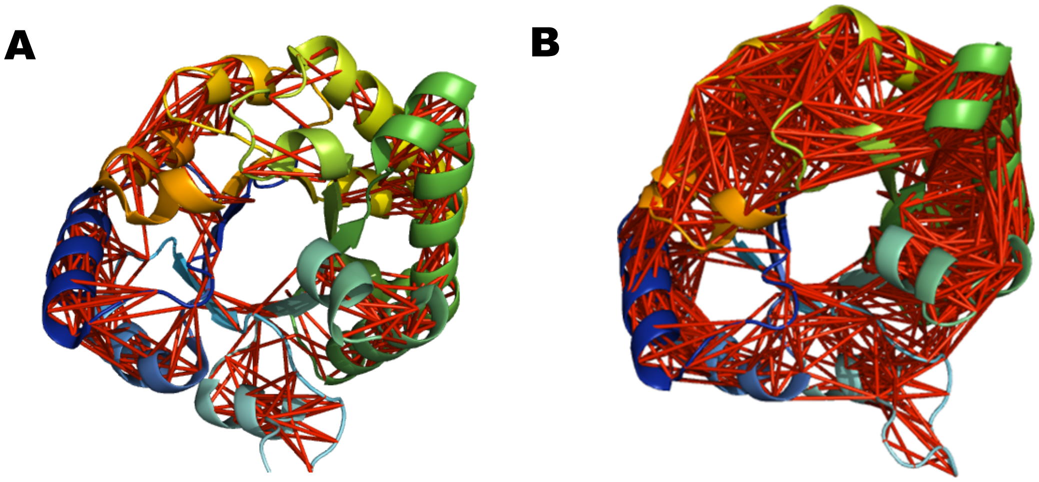

Supplementary Figure 9 – Distribution of distant significant distant correlations in the monomeric form (A) and chain A of the dimeric form (B) of 1N55. We have the top views from the perspective of the C-terminal end, where the respective structures are displayed with the cartoon representation in rainbow (N-terminal in blue, C-terminal in red), and sticks between each pair of residue positions with significant correlations at least 8 Å apart (cf. Methods). The red sticks indicate positive correlations above the score threshold at the 95th percentile rank of the absolute values of the correlations. The oligomeric interface spans the first three β-α secondary structure units (blue to cyan).
