# Supplementary material for: Similarity in Shape Dictates Signature Intrinsic Dynamics Despite No Functional Conservation in TIM Barrel Enzymes
Source: PLoS Comput Biol. 2016 Mar 25;12(3):e1004834. doi: 10.1371/journal.pcbi.1004834 (PMC4807811; doi:10.1371/journal.pcbi.1004834)
Supplement: S1 Table — (PDF) [file pcbi.1004834.s010.pdf]

Supplementary Table 1 – Summary of the five main TBF structures studied.

Enzyme	CATH ID<br><br>Superfamily name	PDB ID/Chain, Domain (Sequence index)	E.C.	Catalytic residues	Substrate and/or metal-ion binding residues	Phosphate binding?[1]
Triosephosphate isomerase	3.20.20.70<br><br>Aldolase Class I	1N55/Chain A, 00 (2-250)	5.3.1.1	From [7]: N11, K13, H95, E167	From 1NEY[63]: A171, I172, G212, L232	From [7]: G173, S213, G234, G235
Chitinase B	3.20.20.80<br><br>Glycosidase	1E15/Chain A01 (3-290, 381-448)	3.2.1.14, 3.2.1.96	From [64]: Y214, D140, D142, E144	From 1E6N[65]: Y10, F51, W97, Y98, Y145, F190, F191, M212, D215, W220, E221, L265, W403	No
Methylaspartate ammonia lyase	3.20.20.120<br><br>Enolase	1KKO/Chain A02 (165-411)	4.3.1.2, 4.2.113	H194, K331	From [66]: Q329, C361, T360, Q172, L384 / Mg2+: D238, E273, D307	No
Transaldolase B	3.20.20.70<br><br>Aldolase Class I	3CWN/ Chain A00 (2-317)	2.2.1.2	From [67]: K132, E96 via water, T156 via water and D17	N35, N154, S176, F178, S226	From [67] & [9]: R228, S226, R181
Glycerophosphodiester phosphodiesterase	3.20.20.190<br><br>Phosphatidylinositol (PI) phosphodiesterase	3CH0/ Chain A00 (0-271)	3.1.4.46	From [68]: H13, H55[2]	H13, R14, E40, D42, H55, E135, K137 / Ca2+: E40, D42, E135	H13, R14, H55 and K137

[1] This refers to sites that bind to phosphate moieties of the substrate, and may or may not have the standard phosphate-binding motif as defined in Nagano (2002).

[2] Reaction involves the phosphate moiety of the substrate, so both the catalytic residues and phosphate-binding residues are the same.
