# Supplementary material for: Similarity in Shape Dictates Signature Intrinsic Dynamics Despite No Functional Conservation in TIM Barrel Enzymes
Source: PLoS Comput Biol. 2016 Mar 25;12(3):e1004834. doi: 10.1371/journal.pcbi.1004834 (PMC4807811; doi:10.1371/journal.pcbi.1004834)
Supplement: S2 Table — (PDF) [file pcbi.1004834.s011.pdf]

Supplementary Table 2 - TBF structures and their additional homologues

Representative structure from superfamily	Homologues
1KKO	1KD0, 3ZVI
1N55	1WYI, 1YPI, 2I9E, 2VEK
1E15	1D2K, 1HKK, 1ITX, 3G6L
3CH0	2O55, 2OOG, 2PZ0, 3L12
3CWN	1F05, 3CQ0, 3HJZ, 3TK7

Only chain A structures used, with domain definition as of CATHv4.0
