# Supplementary material for: Similarity in Shape Dictates Signature Intrinsic Dynamics Despite No Functional Conservation in TIM Barrel Enzymes
Source: PLoS Comput Biol. 2016 Mar 25;12(3):e1004834. doi: 10.1371/journal.pcbi.1004834 (PMC4807811; doi:10.1371/journal.pcbi.1004834)
Supplement: S3 Table — (PDF) [file pcbi.1004834.s012.pdf]

Supplementary Table 3 – Oligomeric forms of the TBF enzyme dataset, as defined by the PDB and PISA.

Representative structure	Biological Assembly (Oligomeric form)	
	Monomeric	Dimeric
1KKO	-	1KKO*, 1KD0*, 3ZVI*
1N55	2VEK	1N55*, 1WYI*, 1YPO*, 2I9E*
1E15	1D2K, 1HKK, 1ITX, 3G6L	1E15*
3CH0	3CH0, 2O55, 2OOG	2PZ0*, 3L12*
3CWN	3HJZ, 3TK7	3CWN*, 1F05*, 3CQ0*
