# Supplementary material for: Similarity in Shape Dictates Signature Intrinsic Dynamics Despite No Functional Conservation in TIM Barrel Enzymes
Source: PLoS Comput Biol. 2016 Mar 25;12(3):e1004834. doi: 10.1371/journal.pcbi.1004834 (PMC4807811; doi:10.1371/journal.pcbi.1004834)
Supplement: S1 Methods — (PDF) [file pcbi.1004834.s013.pdf]

Supplementary Methods

Normalised ideal vectors as defined in the main text (Figure 11).

The normalised ideal vector is a 3*N* vector, where *N* is the number of Cα atoms in the protein. All ideal vectors are normalised by multiplying with the reciprocal of the vector norm. Since the normal modes are orthonormal, the cumulative squared inner products with the ideal vector and the normal modes is one.

The 3N vector contains 3 Cartesian coordinates for each Cα atom position considered in the calculation. For Cα atom position *i* in the collection of SSEs, such as the beta barrel core or the alpha helical bundle, denoted by the subscript α, the normalised ideal vector of the vertical displacement can be described by:

$$\left[\mathbf{z}_{\alpha}^{\text{vertical}}\right]_{\boldsymbol{i}} = \mathbf{a}^{\text{I}} \quad (1)$$

where $\mathbf{a}^{\text{I}}$ is the principal axis of inertia of the whole structure, a 3-vector. For positions in the structure where the Cα atoms are not in the collection of SSEs, this vector is treated as (0,0,0), which is the case for all the normalised ideal vector displacements defined. Thus, $\mathbf{z}^{\text{vertical}}$ is a 3*N* vector that can be projected on to the normal modes of the structure to consider the contribution of only the Cα atoms in the collection of SSEs.

Similarly, the rotation vector of the a single Cα atom in the collection of SSEs is described as:

$$[\mathbf{z}_{\alpha}^{\text{rot}}]_{\boldsymbol{i}} = \mathbf{a}^{\text{I}} \times (s_i - s^{\text{com}}) \quad (2)$$

where the cross-product of the $\mathbf{a}^{\text{I}}$, principal axis of inertia of the structure, and the distance vector between the Cartesian coordinates of position *i* in the SSE collection, s_i and the centre of mass $\mathbf{s}^{\text{com}}$, of the structure produces the idealised rotation displacement vector for that position.

To analyse the normalised ideal vector displacements of the individual SSE in these structures, the vertical displacement vector, similar to (1), of a Cα atom position, *i*, in the *n*th SSE, can be described by:

$$\left[\mathbf{z}_{n}^{\text{vertical}}\right]_{\boldsymbol{i}} = \mathbf{a}_{n}^{\text{I}} \quad (3)$$

where $\mathbf{a}^{\text{I}}_n$ is the vector for the principal axis of inertia of the *n*th SSE.

The individual horizontal displacement vector is the planar translation of the SSE away from the central principal axis of inertia of the main structure. The vector at the *i*th position within the *n*th SSE is defined by the double cross product of the principal axis of inertia of the structure, $\mathbf{a}^{\text{I}}$ with the distance vector between the centre of mass s^{com} of the structure, and of the $s_{\text{n}}^{\text{com}}$:

$$\left[\mathbf{z}_{n}^{\text{horizontal}}\right]_{i} = \mathbf{a}^{\text{I}}\times(\,\mathbf{a}^{\text{I}}\times(s^{\text{com}} - s_{n}^{\text{com}})) \quad (4)$$

The tilting of the SSE describes a swivel about its centre of mass, with the C-terminal and the N-terminal halves of the SSE moving in opposite directions. It is defined by the sliding vector weighted by each Cα atom's distance from the centre of mass using their y-coordinates, as the

principle axis of inertia of the whole structure is aligned to the y-axis in Cartesian coordinate space (Fig 10 in main text).

$$[\mathbf{z}_n^{\text{tilt}}]_i = w_i[\mathbf{z}_n^{\text{horizontal}}]_i \tag{5}$$

where:

$$w_i = y_i - y_n^{\text{com}} \tag{6}$$

The C-terminal bending vector is similar to the tilt with the vectors of the opposite above the centre of mass being weighted by a very small number (ε), in this case 0.000001, to minimise their influence in the overlap.

$$[\mathbf{z}_n^{\text{bend}}]_i = \begin{pmatrix} w_i\ [\mathbf{z}_n^{\text{horizontal}}]_i, \text{for } w_i\ \geq 0 \\ \varepsilon[\mathbf{z}_n^{\text{horizontal}}]_i, \text{for } w_i\ < 0 \end{pmatrix} \tag{7}$$

For the N-terminal bending, the weighting $\mathbf{z}^{\text{bend}}$ is the opposite. Thus, the weighting is applied on the vectors below the center of mass of the secondary structure, while the vectors above are multiplied by ε.
